# Supplementary material for: B Cell Diversification Is Uncoupled from SAP-Mediated Selection Forces in Chronic Germinal Centers within Peyer’s Patches
Source: Cell Rep. 2020 Feb 11;30(6):1910–1922.e5. doi: 10.1016/j.celrep.2020.01.032 (PMC7016508; doi:10.1016/j.celrep.2020.01.032)
Supplement: Document S1. Figures S1–S6 and Table S1 [file mmc1.pdf]

Cell Reports, Volume *30*

Supplemental Information

B Cell Diversification Is Uncoupled

from SAP-Mediated Selection Forces in Chronic

Germinal Centers within Peyer's Patches

Adi Biram, Eitan Winter, Alice E. Denton, Irina Zaretsky, Bareket Dassa, Mats Bemark, Michelle A. Linterman, Gur Yaari, and Ziv Shulman

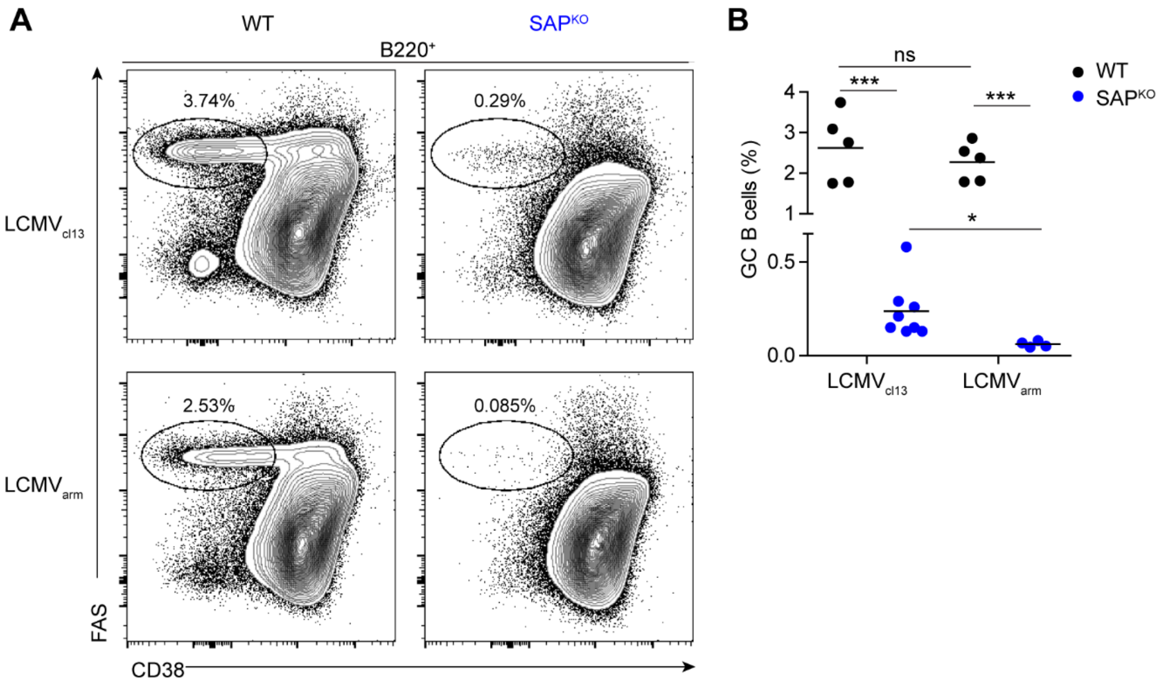

Figure S1. GC formation in the spleen in response to acute and chronic LCMV infection, Related to figure 2. (A) Representative flow cytometry plots showing GC frequency ($B220^+$ FAS^+ $CD38^-$) in the spleen of WT and SAP^{KO} mice, 30 days following intravenous infection with $LCMV_{cl13}$ or $LCMV_{arm}$. **(B)** Quantification of GC frequencies analyzed as in (A). * $P<0.05$, *** $P<0.0001$, one-way ANOVA with Bonferroni posttest. ns, not significant.

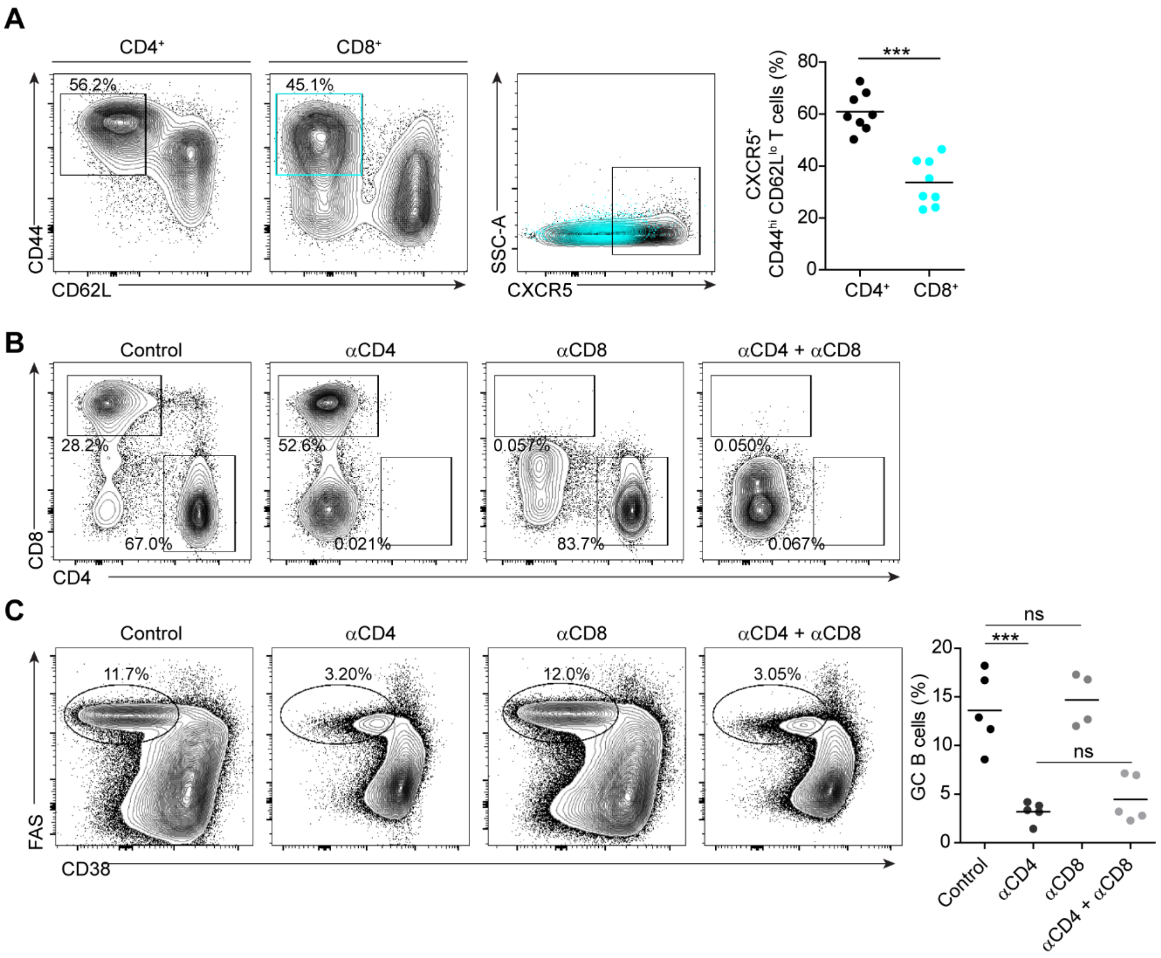

Figure S2. CD8 T cell depletion does not affect GC frequency, Related to figure 3. (A) Representative flow cytometry plots showing gating on activated T cells (CD44^{hi} CD62L^{lo}) and CXCR5 expression on CD4^{+} or CD8^{+} activated T cells. Quantification is summarized in the graph. **(B)** Representative flow cytometry plots showing the CD4^{+} and CD8^{+} T cell populations (gated on CD3^{+}) following intravenous administration of 200µg or 100µg depleting antibodies for CD4 and CD8 depletion, respectively. **(C)** Representative flow cytometry plots showing GC frequency following the different treatments as described in B. Quantification is shown in the graph. *** P<0.0001, two-tailed Student's t test in (A). One-way ANOVA with Bonferroni posttest in (C). ns, not significant.

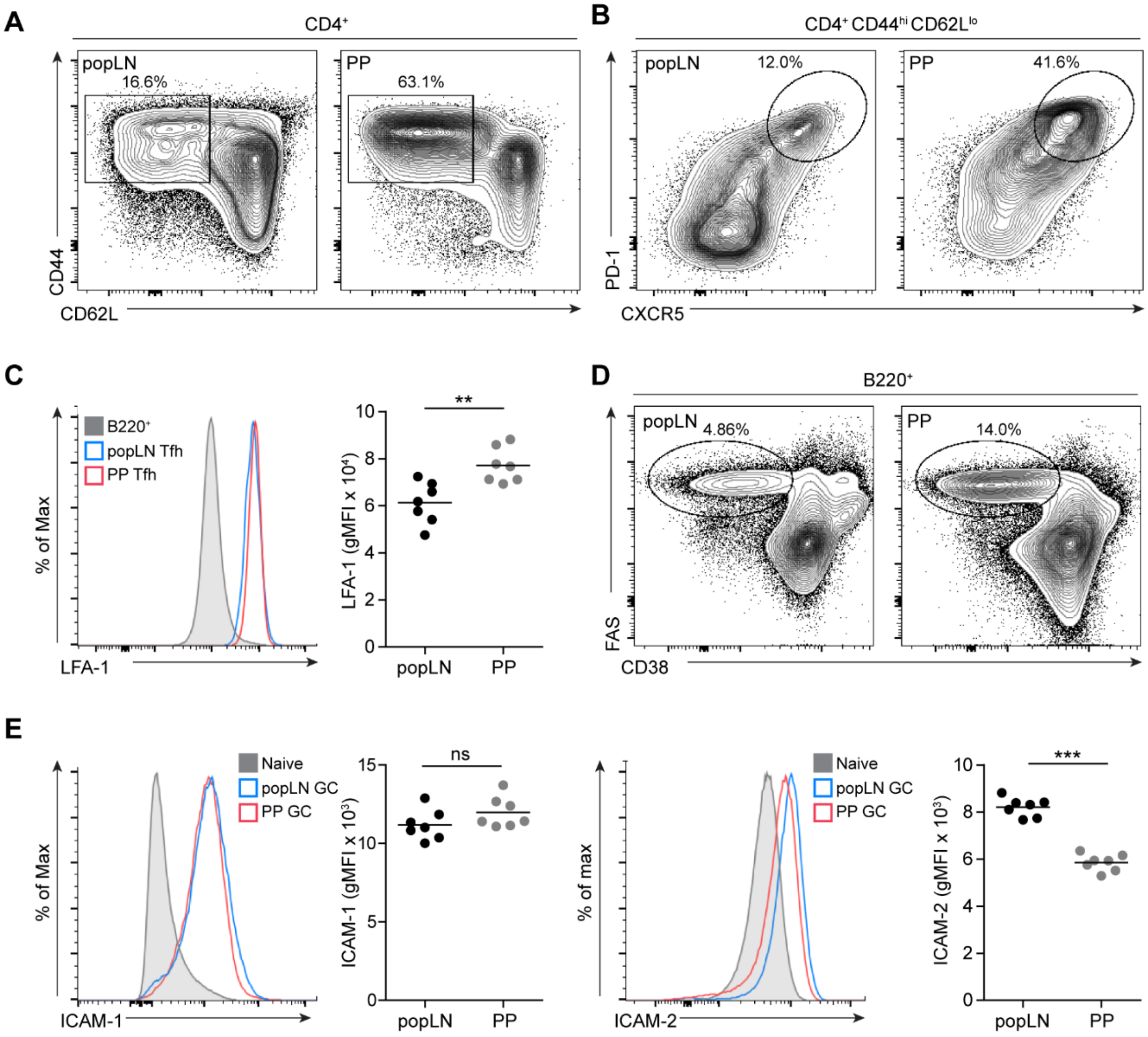

Figure S3. LFA-1 and ICAM expression on Tfh and GC B cells in popLNs and PPs, Related to figure 3. (A) Representative flow cytometry plots showing gating of activated T cells ($CD44^{hi}$ $CD62L^{lo}$) in popLN and PP of WT mice, 7 days after NP-OVA intra-footpad immunization. **(B)** Representative flow cytometry plots showing Tfh cell population ($CXCR5^{hi}$ $PD\text{-}1^{hi}$) in T cells gated as in (A). **(C)** Representative histogram showing LFA-1 expression in Tfh cells gated as in (B). Graph shows quantification. **(D)** Representative flow cytometry plots showing gating of GC B cells ($B220^{+}$ FAS^{+} $CD38^{-}$) in PPs and immunized popLNs, as described in (A). **(E)** Representative histograms and graphs showing ICAM-1 and ICAM-2 expression in GC B cells gated as in (D). ** P<0.001, *** P<0.0001, two-tailed Student's t test; ns, not significant.

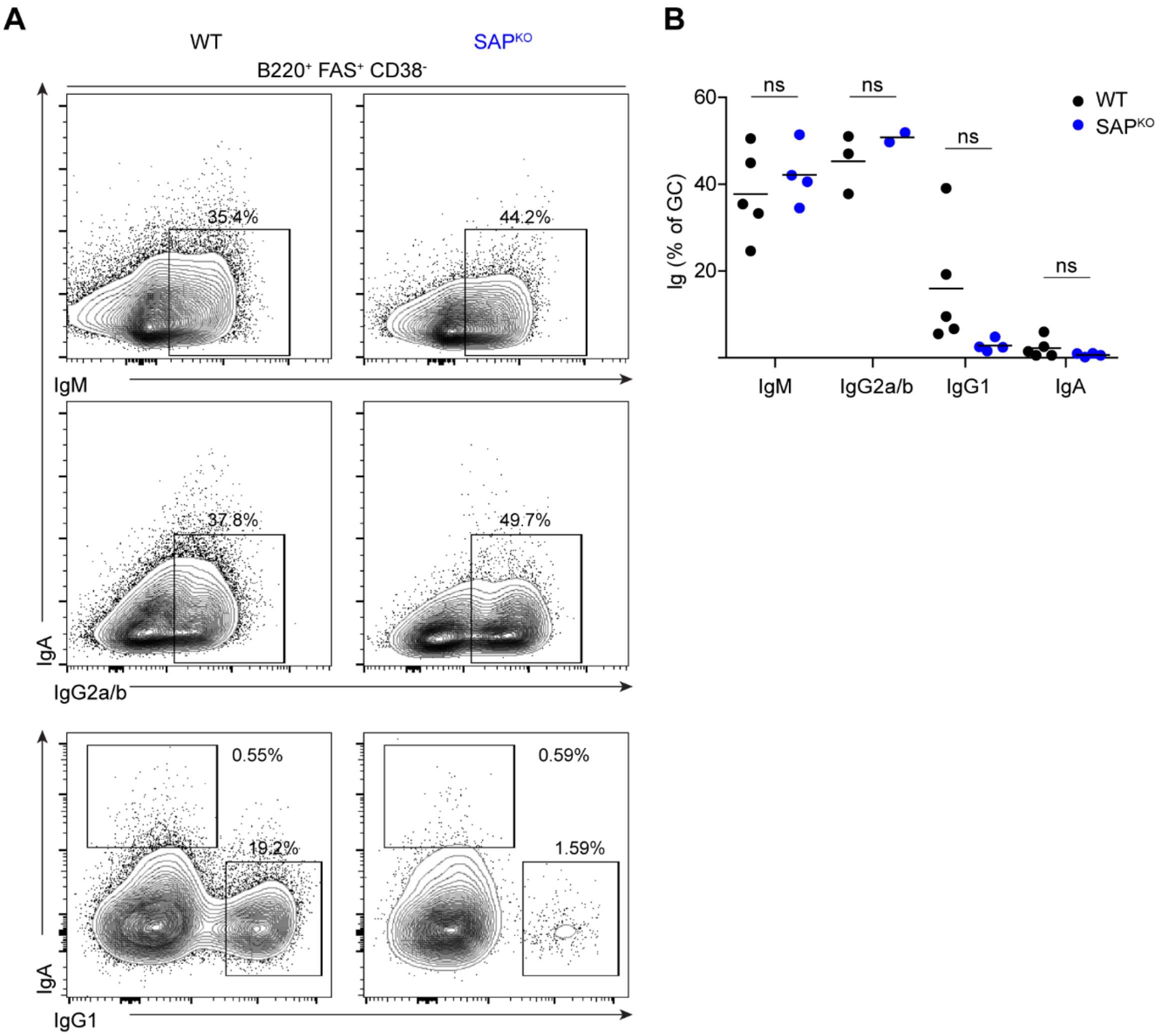

Figure S4. Ig isotypes of GC B cells in the mediastinal LN of SAP^{KO} mice following influenza infection, Related to figure 2. (A,B) Representative flow cytometry plots (A) and summary graph (B) showing IgM, IgG2a/b, IgA and IgG1 staining of germinal center B cells (FAS^{+} CD38^{-}) derived from mediastinal LNs of influenza (x31) infected WT and SAP^{KO} mice, 14 days after intranasal infection. One-way ANOVA with Bonferroni posttest; ns, not significant.

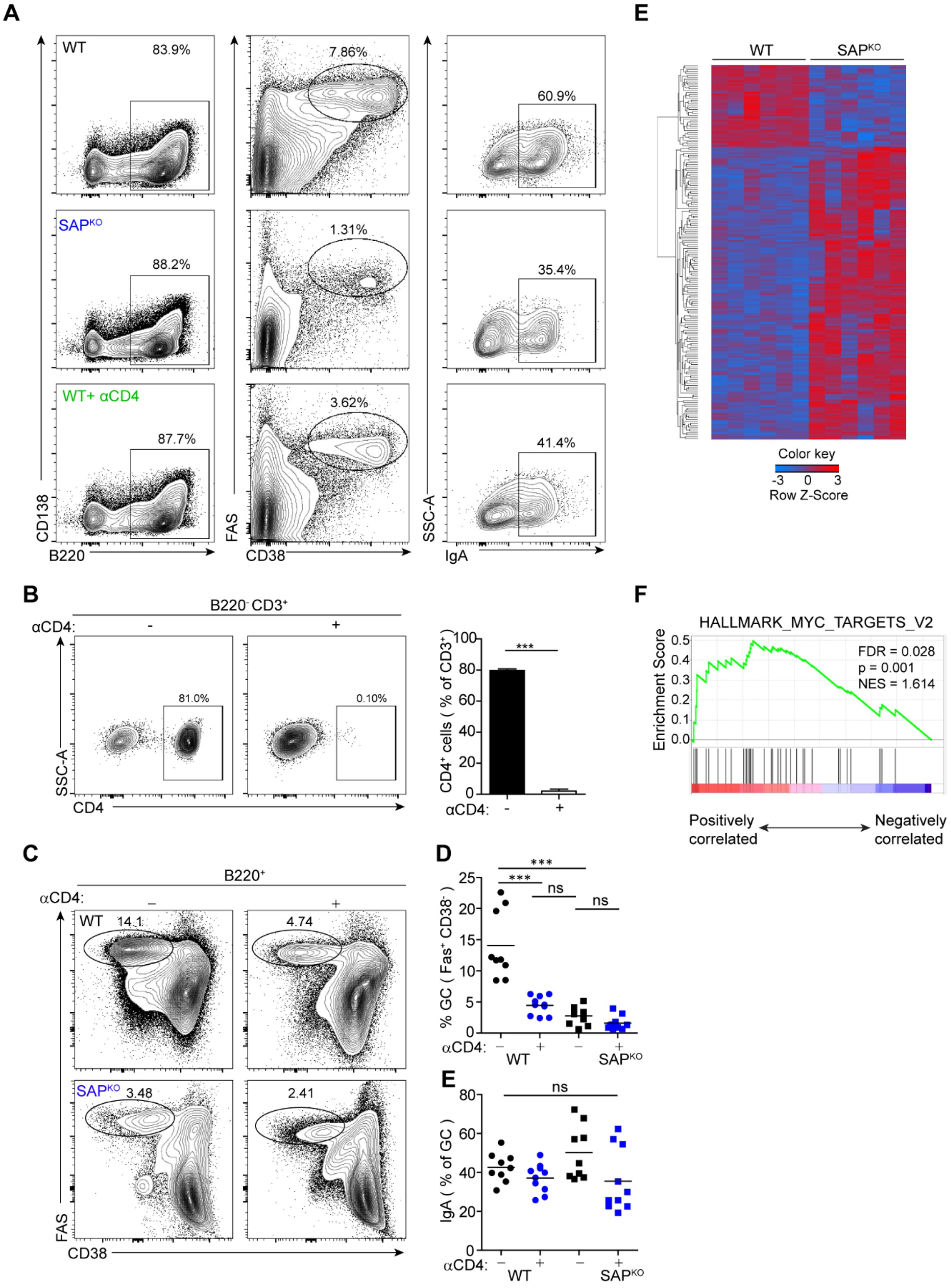

A
WT
83.9%
7.86%
60.9%
SAPKO
88.2%
1.31%
35.4%
WT+ αCD4
87.7%
3.62%
41.4%
CD138
B220
FAS
CD38
SSC-A
IgA
E
WT
SAPKO
Color key
-3 0 3
Row Z-Score
B
B220- CD3+
αCD4:
81.0%
0.10%
SSC-A
CD4
CD4+ cells (% of CD3+)
***
C
B220+
WT 14.1
4.74
SAPKO 3.48
2.41
FAS
CD38
D
% GC (Fas+ CD38-)
***
ns
WT
SAPKO
IgA (% of GC)
F
HALLMARK_MYC_TARGETS_V2
Enrichment Score
FDR = 0.028
p = 0.001
NES = 1.614
Positively correlated
Negatively correlated

Figure S5. Gating strategy for PP IgA GC single-cell sorting for *Igh* sequencing, T cell depletion and GC B cell transcriptomic analysis, Related to figure 5. (A) Representative flow cytometry plots showing the strategy for single-cell sorting of $B220^{+}$ FAS^{+} $CD38^{-}$ IgA^{+} cells from a single PP of WT, SAP-deficient, and [WT + αCD4] mice. [WT + αCD4] mice were treated with an αCD4 depleting antibody for 14 days. **(B)** Representative flow cytometry plots showing the $CD4^{+}$ cell population in PPs of WT mice treated with 200μg αCD4 depleting antibody, 4 days after antibody administration. Graph shows quantification of $CD4^{+}$ cell number as in (A). **(C)** Representative flow cytometry plots showing GC frequency in WT and SAP-deficient PPs following T cell depletion. **(D)** Quantification of GC frequency, as presented in (C). **(E)** Frequency of IgA class switch recombination in GC B cells of WT and SAP-deficient PPs following αCD4 treatment. Data are pooled from three independent experiments with three mice in each experiment. Each dot represents a single mouse; line represents the mean. * $P<0.05$, *** $P<0.0001$, one way ANOVA with Bonferroni posttest in (D,E) and two-tailed Student's t test in (B). ns, not significant. **(F)** Heat map showing expression levels of differentially expressed genes (n=6 in each group), as analyzed by UTAP pipeline using a cutoff of: padj <= 0.05, |log2FoldChange| >= 1, and BaseMean >= 5. Expression levels of genes (log2 normalized, rld) were scaled and clustered by Hierarchical clustering (Euclidean, average linkage) and visualized using Partek Genomics Suite. **(G)** Gene set enrichment plot for Myc pathway target genes from the hallmark gene sets. FDR, Flase discovery rate; p, p-value; NES, normalized enrichment score. In H,I, six mice were used in each group (n=6).

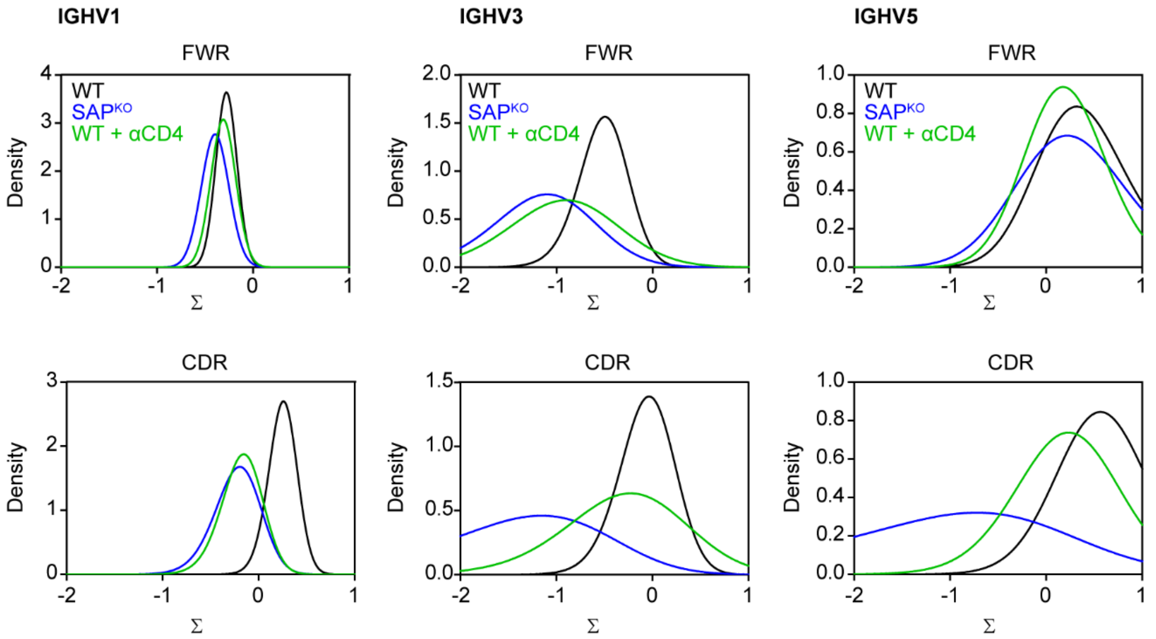

Figure S6. Estimation of selection in single V families shared among WT and SAP^{KO} Peyer's patch germinal center B cells, Related to figure 5. Graphs showing the density of selection strength for variable sequences of IGHV1, IGHV3 and IGHV5 within the framework (FWR) or complementary determining region (CDR) that were detected in WT, SAP^{KO} and αCD4 treated mice. Selection was estimated using BASELINe focused test with the MK RS5NF mutability model, FDR<0.05.

Primer name	Primer sequence 5'-3'
IL4R- F	GAGTGGAGTCCTAGCATCACG
IL4R-R	CAGTGGAAGGCGCTGTATC
IL21R-F	GGAGTGACCCCGTCATCTT
IL21R-R	AGGAGCAGCAGCATGTGAG
Stat3-F	GTTCCTGGCACCTTGGATT
Stat3-R	CAACGTGGCATGTGACTCTT
Stat6-F	GTCCATGAGTGTACTGCCATCT
Stat6-R	GGCATGGTTATCTGGCTCAT
Bcl6-F	GAACTGTATGCAGATTCCAGTCA
Bcl6-R	ACTGTCCTCTTGTAATCCTTCCA
HPRT-F	GTTAAGCAGTACAGCCCCAAA
HPRT-R	AGGGCATATCCAACAACAAACTT

Table S1. List of primers used for qPCR, Related to figure 4.
