# Supplementary material for: B Cell Diversification Is Uncoupled from SAP-Mediated Selection Forces in Chronic Germinal Centers within Peyer’s Patches
Source: Cell Rep. 2020 Feb 11;30(6):1910–1922.e5. doi: 10.1016/j.celrep.2020.01.032 (PMC7016508; doi:10.1016/j.celrep.2020.01.032)
Supplement: Document S2. Article plus Supplemental Information [file mmc2.pdf]

Cell Reports

Article

B Cell Diversification Is Uncoupled from SAP-Mediated Selection Forces in Chronic Germinal Centers within Peyer's Patches

Graphical Abstract

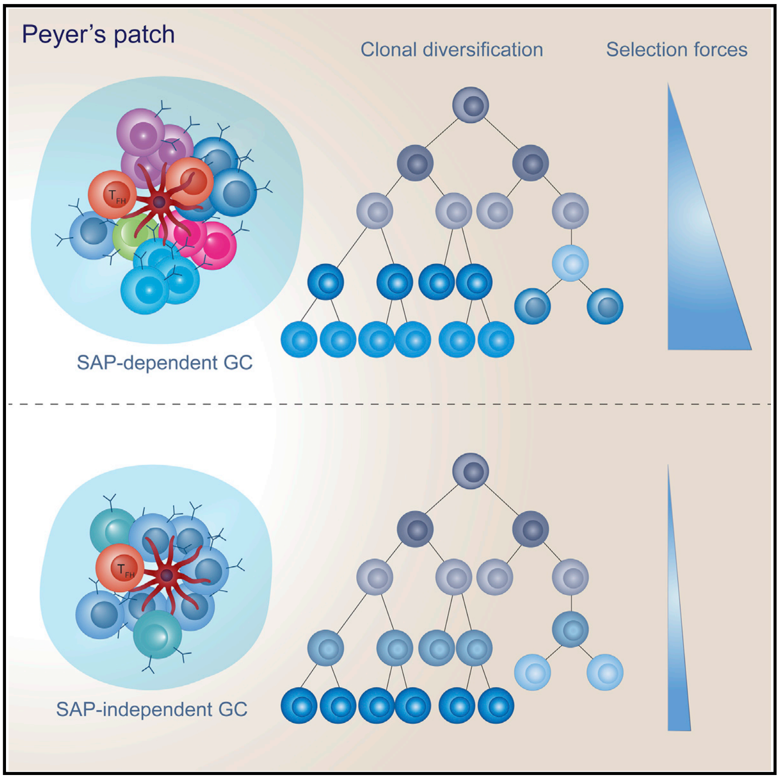

Highlights

- Chronic germinal centers in Peyer's patches are formed in SAP-deficient mice
- SAP-independent germinal centers arise in response to influenza infection
- Few highly diversified clones dominate the SAP-independent germinal centers
- Germinal center B cells in SAP-deficient mice are subjected to mild selection forces

Authors

Adi Biram, Eitan Winter, Alice E. Denton, ..., Michelle A. Linterman, Gur Yaari, Ziv Shulman

Correspondence

ziv.shulman@weizmann.ac.il

In Brief

SAP is required for proper T cell help in germinal centers (GCs). Biram et al. show that SAP-independent GCs are formed within Peyer's patches. These GCs host highly diversified clones that are subjected to mild selection forces, demonstrating that clonal diversification can be uncoupled from clonal selection in chronic GCs.

Biram et al., 2020, Cell Reports *30*, 1910–1922
February 11, 2020 © 2020 The Authors.
https://doi.org/10.1016/j.celrep.2020.01.032

CellPress

OPEN
ACCESS
CellPress

Cell Reports
Article

B Cell Diversification Is Uncoupled from SAP-Mediated Selection Forces in Chronic Germinal Centers within Peyer's Patches

Adi Biram,[1] Eitan Winter,[2] Alice E. Denton,[3] Irina Zaretsky,[1] Bareket Dassa,[4] Mats Bemark,[5] Michelle A. Linterman,[3] Gur Yaari,[2] and Ziv Shulman[1,6,*]
[1]Department of Immunology, Weizmann Institute of Science, Rehovot 7610001, Israel
[2]Faculty of Engineering, Bar Ilan University, Ramat Gan 52900, Israel
[3]Laboratory of Lymphocyte Signaling and Development, Babraham Institute, Cambridge CB22 3AT, UK
[4]Department of Life Science Core Facilities, Weizmann Institute of Science, Rehovot 7610001, Israel
[5]Department of Microbiology and Immunology, Institute of Biomedicine, University of Gothenburg, Gothenburg SE-405 30, Sweden
[6]Lead Contact
*Correspondence: ziv.shulman@weizmann.ac.il
https://doi.org/10.1016/j.celrep.2020.01.032

SUMMARY

Antibodies secreted within the intestinal tract provide protection from the invasion of microbes into the host tissues. Germinal center (GC) formation in lymph nodes and spleen strictly requires SLAM-associated protein (SAP)-mediated T cell functions; however, it is not known whether this mechanism plays a similar role in mucosal-associated lymphoid tissues. Here, we find that in Peyer's patches (PPs), SAP-mediated T cell help is required for promoting B cell selection in GCs, but not for clonal diversification. PPs of SAP-deficient mice host chronic GCs that are absent in T cell-deficient mice. GC B cells in SAP-deficient mice express AID and Bcl6 and generate plasma cells in proportion to the GC size. Single-cell IgA sequencing analysis reveals that these mice host few diversified clones that were subjected to mild selection forces. These findings demonstrate that T cell-derived help to B cells in PPs includes SAP-dependent and SAP-independent functions.

INTRODUCTION

The clearance of invading microbes and the establishment of enduring protection from harmful pathogens depends on B cell differentiation into plasma cells (PCs) that secrete high-affinity antibodies. These cells are generated in microanatomical sites, known as germinal centers (GCs), which emerge in lymphoid organs primarily in response to vaccination or pathogen invasion (Victora and Nussenzweig, 2012). In these sites, B cells that express antigen-specific B cell receptors (BCRs) undergo clonal diversification by somatic hypermutation (SHM) and affinity-based clonal selection (Berek et al., 1991; Eisen and Siskind, 1964), and subsequently emerge as either memory or antibody-secreting cells (Corcoran and Tarlinton, 2016). Both entry into the GC and the selection of B cells bearing high-affinity BCR variants are regulated by T follicular helper (Tfh) cells, specialized $CD4^+$ T cells that physically interact with B cells and distinguish high- versus low-affinity clones, based on their capacity to take up and present surface antigens (Schwickert et al., 2011; Victora et al., 2010; Vinuesa and Cyster, 2011). Although this process was intensively studied in lymph nodes (LNs) and spleen in response to immunization, it is not clear whether Tfh cells play a similar role in chronic GC responses within intestinal lymphoid organs.

The composition of the gut microbiota is modulated by a relatively stable PC population that resides in the gut and secretes immunoglobulin A (IgA) antibodies that bind various specific bacterial epitopes and maintain the homeostatic balance between the host and commensal bacteria (Gibbons and Spencer, 2011; Hapfelmeier et al., 2010; Lindner et al., 2012). Class switching to the IgA isotype takes place within Peyer's patches (PPs) (Craig and Cebra, 1971), predominantly in an area known as the subepithelial dome (SED) (Biram et al., 2019a; Reboldi et al., 2016). As opposed to class switch recombination (CSR) of B cells to IgG1 within draining LNs and spleen in response to immunization or microbe invasion, switching to the IgA isotype in PPs can take place in the absence of T cell-derived signals (Bergqvist et al., 2006; Macpherson et al., 2000; Mombaerts et al., 1994). It remains unknown whether other B cell functions in chronic GCs, such as clonal diversification and affinity-based selection, require T cell help.

The BCR plays a dual role during cognate antigen recognition; it propagates signal transduction and mediates the uptake of antigens for processing and presentation on surface major histocompatibility complex class II (MHC class II) molecules to Tfh cells (Kometani and Kurosaki, 2015; Victora and Nussenzweig, 2012). Surface and secreted help signals from T cells to B cells are essential for GC formation and function in LNs and spleen (Vinuesa and Cyster, 2011). Nonetheless, several studies that examined GCs in mucosal tissues provided evidence that challenges this model. RAG-deficient mice that were crossed to transgenic strains carrying a single and non-cognate T cell receptor (TCR) and BCR were able to form GC structures in PPs (Bemark et al., 2000). Furthermore, it was found that BCR-deficient B cells that express the Epstein-Barr virus (EBV) protein LMP2A were unable to form GCs in the spleen in response to immunization. Although antigen uptake and presentation cannot take place in the absence

This is an open access article under the CC BY license (http://creativecommons.org/licenses/by/4.0/).

of a BCR, GC structures were detected in the gut-associated lymphoid tissues (GALTs) of LMP2A transgenic mice (Casola et al., 2004). Moreover, it was demonstrated that in transgenic mice in which nearly all of the B cells are specific for a single antigen, GCs were formed in PPs, despite the fact that these B cells never encountered their cognate antigen (Casola et al., 2004; Yeap et al., 2015). In addition, highly mutated and expanded clones specific for lipopolysaccharides (LPS; an antigen that drives T cell-independent responses) were found in the human gut, suggesting that these cells originated from the GC response, although they were unable to present peptides to T cells (Kauffman et al., 2016). Nevertheless, T cells play a critical role in these responses, since GC formation in PPs and mesenteric LNs (mLNs) does not take place in T cell- and CD40-deficient mice (Bergqvist et al., 2006; Casola et al., 2004; Lindner et al., 2012; Macpherson et al., 2000). These observations indicate that the T cell help mechanisms in chronic GC reactions in PPs do not necessarily play an identical role to those in GC responses that emerge within LNs in response to immunization. Accordingly, it seems that a more complex B cell response takes place in the gut, which cannot be simply classified according to the traditional T cell-dependent versus independent dichotomy.

Tfh cells express high levels of the cytoplasmic signaling lymphocyte activation molecule (SLAM)-associated protein (SAP), encoded by the *Sh2d1a* gene (Crotty et al., 2003; McCausland et al., 2007; Schwartzberg et al., 2009). SAP functions as an inhibitor of negative signals by competing with SHP1 for the binding of the immunoreceptor tyrosine-based switch motifs (ITSM) domain in the cytoplasmic tail of Ly108, a member of the SLAM receptor family (Chu et al., 2014; Kageyama et al., 2012). This adaptor is critical for Tfh cell functions, as T cells deficient in SAP are unable to promote GC formation as a result of defects in their development and in their ability to deliver proper T cell help signals to B cells (Biram et al., 2019b; Cannons et al., 2006, 2010; Qi et al., 2008; Schwartzberg et al., 2009). Furthermore, it was shown that T cell functions and SAP expression are required for GC maintenance in the spleen and LNs (Jones et al., 2016; Zhong and Veillette, 2013). Mucosal lymphoid organs such as PPs and mLNs perpetually collect bacteria-derived antigens, and therefore constitutively host GC reactions (Reboldi and Cyster, 2016). It remained to be determined whether SAP-mediated T cell help plays a role in these chronic GCs during homeostasis similar to that observed in inducible GC reactions in peripheral LNs.

In the present study, we examined the role of SAP in regulating chronic GC reactions that form in response to commensal bacteria- and dietary-derived antigens. We found that SAP is not required for the formation of GCs in PPs and for clonal diversification of B cells; however, SAP-mediated T cell help is essential for proper B cell selection within chronic GCs in PPs. We conclude that T cell help to B cells in PP GCs involves both SAP-dependent and SAP-independent functions.

RESULTS

SAP-Deficient Mice Host Small GCs within PPs

SAP-mediated T cell help is essential for mounting a T cell-dependent immune response in draining LNs and spleen in response to immunization or microbe invasion, but it is not known whether this adaptor protein regulates chronic immune responses in the gut. To examine the role of SAP in GC formation in PPs, we imaged GCs of wild-type (WT), SAP knockout (SAP^{KO}), and T cell-deficient mice ($TCR\alpha^{KO}$) by deep scanning of intact organs using two-photon laser scanning microscopy (TPLSM). In PPs, the enzyme activation-induced cytidine deaminase (AID) is expressed primarily by GC B cells and to a lesser extent by activated B cells located within the SED (Biram et al., 2019a; Reboldi et al., 2016). To clearly visualize GC structures in SAP- and TCRα-deficient mice, we crossed these strains to mice that express Cre recombinase under the AID promoter together with a conditional tdTomato reporter cassette ($Aicda^{Cre/+}$ $Rosa26^{Stop\text{-}tdTomato/+}$). In these mice, tdTomato is upregulated by cells that express AID or previously expressed AID (Rommel et al., 2013).

We examined GC formation in popliteal LNs of the AID reporter mice in response to subcutaneous immunization with 4-hydroxy-3-nitrophenyl acetyl (NP) conjugated to ovalbumin (NP-OVA) in alum. As expected, 7 days after immunization, GC structures were evident in the LNs of WT, but not in SAP- or TCRα-deficient mice (Figure 1A). Close analysis of the LNs from either SAP- or TCRα-deficient immunized mice revealed that tdTomato-expressing B cells were scattered throughout the LN cortex, demonstrating that T cell help is essential for GC formation but not for initial AID expression (Figure 1A). Similar analysis of PPs and mesenteric LNs derived from these WT mice, which host B cell responses to commensal bacteria- and food-derived antigens, revealed clear GC structures (Figures 1B–1E). However, in sharp contrast to the defect observed in the LNs of SAP-deficient mice, GCs were clearly observed in the PPs and mLNs of these mice. These GCs were T cell dependent, as no GC structures were detected in the PPs and mLNs of TCRα-deficient mice (Figures 1B–1E). The $AID^{Cre/+}$ $Rosa26^{Stop\text{-}tdTomato/+}$ strain is a fate reporter mouse that does not necessarily identify cells that currently express AID and engage in the GC reaction. To examine whether AID is specifically expressed in the GCs of SAP-deficient mice, we imaged PPs derived from an AID-GFP reporter strain (Rommel et al., 2013). To this end, these mice were crossed to SAP- or TCRα-deficient mice and their PPs were subjected to whole LN scan by TPLSM. This analysis revealed clusters of AID-expressing B cells in SAP-deficient mice that were smaller compared to those in AID-GFP WT control mice (Figures 1F and 1G). Miniscule GC-like structures were also detected in the TCRα-deficient AID-GFP mice, although these were significantly smaller than the ones observed in WT and SAP-deficient mice (Figure 1G). We conclude, therefore, that SAP is not essential for the generation of GC structures that contain AID-expressing B cells in PPs.

GC B Cells in SAP-Deficient Mice Express Typical Markers

To further examine whether SAP-deficient mice host conventional GCs, we stained popliteal LN and PP cells with antibodies that detect typical GC markers (e.g., $CD38^{-}$, FAS^{+}). As expected, in WT mice, GCs were generated in response to immunization with NP-OVA in alum, whereas no GCs were detected in LNs derived from SAP- or TCRα-deficient mice (Figures 2A and

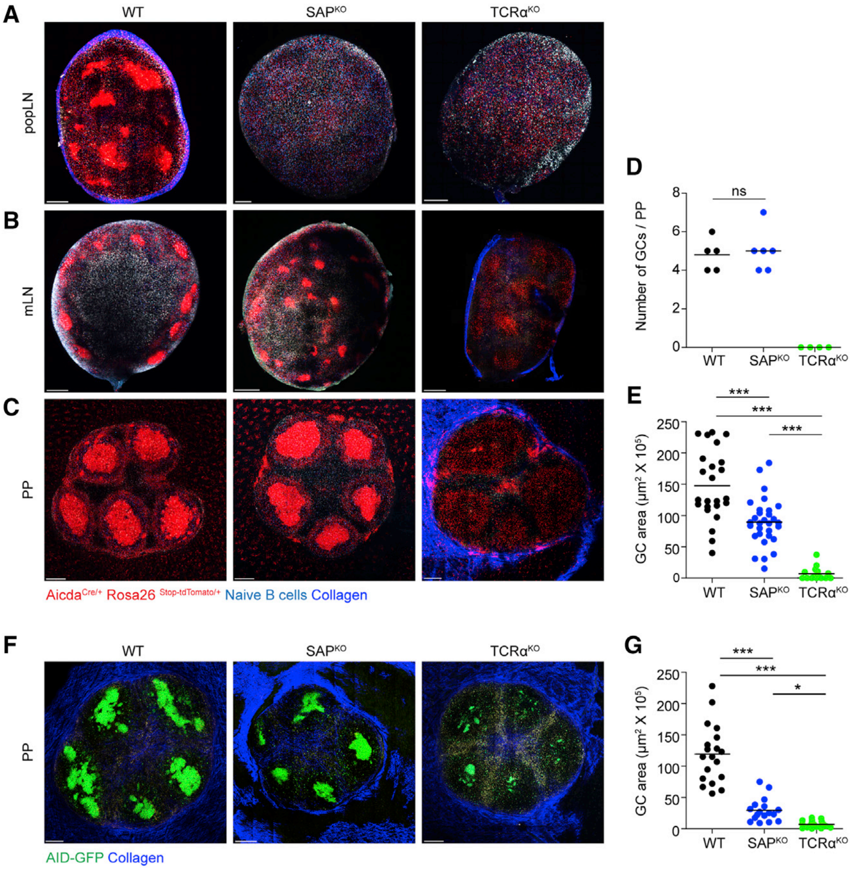

Figure 1. Small GC Structures Are Formed in PPs of SAP-Deficient Mice

(A) TPLSM images of popliteal LNs derived from $AID^{Cre/+}$ $Rosa26^{Stop\text{-}tdTomato/+}$ WT, SAP^{KO}, or $TCR\alpha^{KO}$ mice 7 days after intra-footpad NP-OVA immunization. One day before imaging, naive CFP B cells were transferred to the mice. Scale bar, 200 μm.

(B and C) TPLSM images of mesenteric LNs (B) and PPs (C). Scale bar, 200 μm.

(D) Number of GC structures per PP in WT, SAP^{KO}, and $TCR\alpha^{KO}$ mice.

(E) Quantification of GC area of TPLSM images of the mice as shown in (C).

(F) TPLSM images of PPs derived from AID-GFP WT, SAP^{KO}, or $TCR\alpha^{KO}$ mice. Scale bar, 200 μm.

(G) Quantification of GC area from TPLSM images of the mice as shown in (F).

Data are pooled from three independent experiments with two mice in each experiment. Each dot in (D) represents a single PP; each dot in (E) and (G) represents a single GC structure; line represents the mean. $^{*}p < 0.05$, $^{***}p < 0.0001$, one-way ANOVA with Bonferroni posttest. ns, not significant.

2B). In addition, no GC cells were detected in PPs derived from $TCR\alpha^{KO}$ mice; however, significant frequencies of GC cells were detected in the PPs of unimmunized SAP-deficient mice—3.4-fold lower than in WT (Figures 2A and 2C). In addition, GCs in the PPs of SAP-deficient mice showed normal distribution between the dark zone (DZ) and light zone (LZ) compartments (Figure 2D). Bcl6 is a key transcription factor that is essential for reprogramming activated B cells to differentiate into GC B cells (Basso and Dalla-Favera, 2012). B cells from WT and SAP-deficient mice expressed similar levels of mRNA transcripts and protein of Bcl6 (Figures 2E and 2F). These findings indicate that the GC structures observed in the PPs of SAP-deficient mice are bona fide GCs.

Many chronic GCs are found within PPs, and therefore it is difficult to discern whether these GCs are driven by specific gut-derived antigens. To examine whether SAP is required for pathogen-induced GC formation within other mucosal tissues, we infected WT, SAP-, and TCRα-deficient mice intranasally with an influenza A virus (×31), and examined the draining mediastinal LNs of the lungs by flow cytometry and immunofluorescence staining after 14 days. In response to viral infection, GC B cells (e.g., $Ki67^{+}$, $Bcl6^{+}$) were detected in the mediastinal LNs of WT mice, but not in TCRα-deficient mice, demonstrating that T cells are essential for GC formation in this organ as well (Figures 2G and 2H). Conversely, a clear GC cell population was detected in the mediastinal LNs of infected SAP-deficient mice; however, this GC population was 2.4-fold smaller compared to that observed in WT mice (Figures 2G and 2H). Thus, we conclude that similar to chronic immune responses in gut lymphoid organs, SAP is not essential for GC formation in response to specific viral infection within the lung-draining LNs.

To gain additional insight into the role of SAP in viral infection, we examined whether chronic exposure to immune stimulation promotes GC formation in SAP-deficient mice. It was previously demonstrated that GC formation was severely defective in chronically infected SAP-deficient mice; however, a very small GC B cell population was observed (Crotty et al., 2006). To further investigate this result, we repeated this specific experiment and compared GC formation in SAP-deficient mice in response to either chronic lymphocytic choriomeningitis virus ($LCMV_{cl13}$) or acute ($LCMV_{arm}$) infection. We detected a small population of GC B cells (9.8-fold less than WT) in $LCMV_{cl13}$-infected mice 30 days after infection, whereas GC B cells were not detected in response to administration of the acute virus (Figure S1). We conclude that chronic exposure to antigen may promote the formation of GC B cells to some extent independently of SAP functions.

The Frequency of Tfh Cells in PPs Is Proportional to GC Size

It was previously shown that in SAP-deficient mice, Tfh cell formation is severely impaired (Schwartzberg et al., 2009). We examined using flow cytometry analysis whether sufficient Tfh cells are formed in the PPs of SAP-deficient mice. We found that the frequency of activated Th cells ($CD44^{hi}$ $CD62L^{lo}$) was similar between WT and SAP-deficient mice (Figures 3A and 3C); however, the frequency of Tfh cells, based on CXCR5 and PD-1 marker expression, was 4-fold lower compared to WT mice (Figures 3B and 3D). Nonetheless, a clear population of

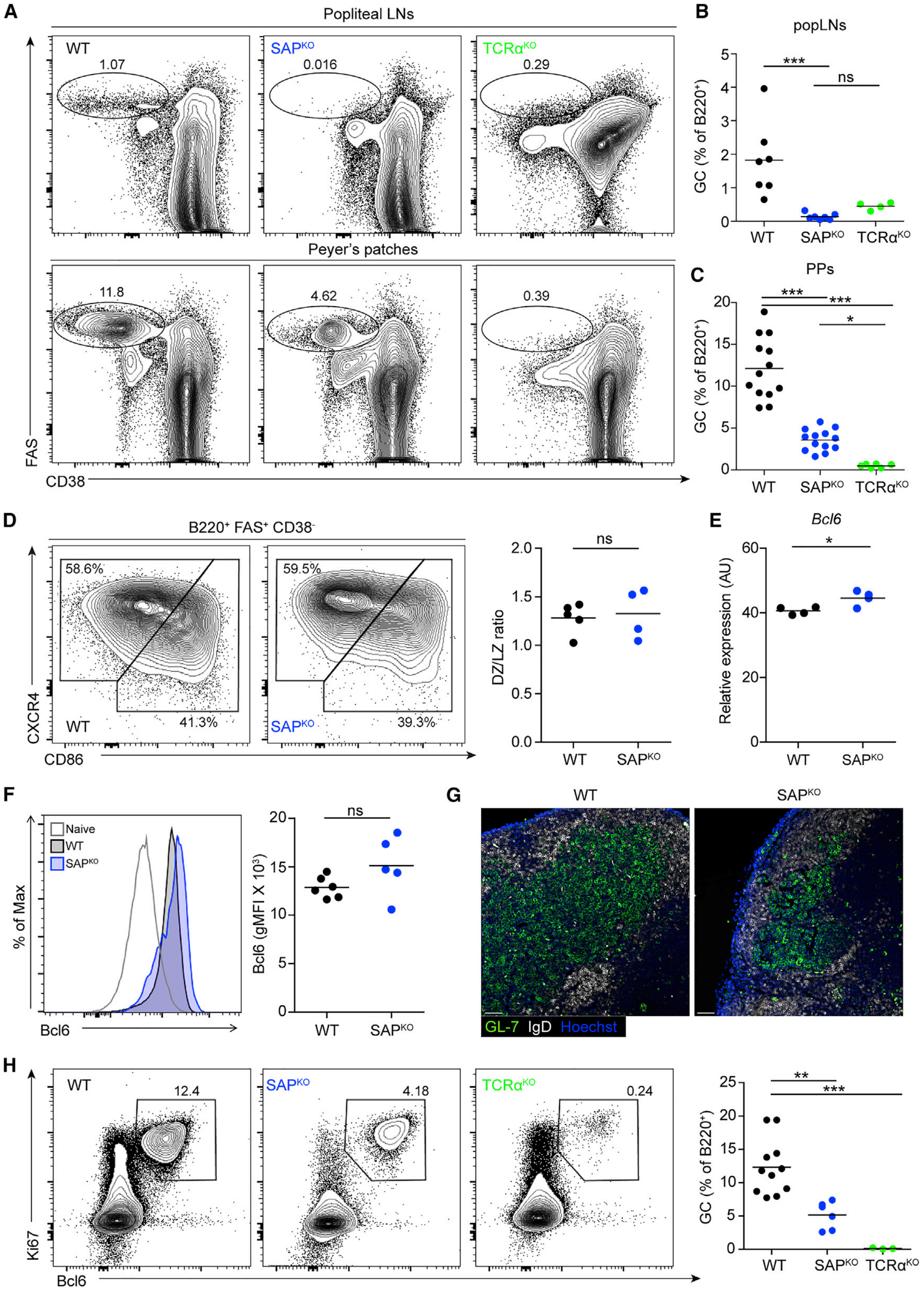

Figure 2. GC B Cells in PPs of SAP-Deficient Mice Express Typical Markers

(A) Representative flow cytometry plots showing GC B cells ($CD38^{-}$ FAS^{+}) in popliteal LNs (top panel) 7 days after intra-footpad NP-OVA immunization and in PPs of WT, SAP^{KO}, or $TCR\alpha^{KO}$ mice.

(legend continued on next page)

$CD4^+$ cells that expressed high levels of CXCR5, PD-1, and the Tfh transcription factor Bcl6 was present in the PPs of SAP-deficient mice (Figure 3E). The GC B cell:Tfh ratio was similar between SAP-deficient and WT mice, suggesting that sufficient numbers of T cells were available for each GC B cell (Figure 3F). It was previously suggested that $CXCR5^+$ CD8 T cells can provide T cell help to B cells (Shan et al., 2017); however, the depletion of CD8 T cells in PPs had no measurable effect on the GC size, whereas depletion of CD4 or total T cells had a dramatic effect (Figure S2). Thus, optimal generation of Tfh cells in PPs depends on the expression of SAP in CD4 T cells; nonetheless, their proportion relative to GC B cells is not perturbed in SAP^{KO} mice.

Next, we examined whether B cells in the GCs of SAP-deficient mice express molecules that mediate the delivery of T cell help and proliferation-induced signals (Biram et al., 2019b; Liu et al., 2015; Zaretsky et al., 2017). Although the expression levels of some adhesion molecules on GC T and B cells in lymph nodes were slightly different in the PPs (Figure S3), we found that the receptors controlling T cell-B cell interactions and delivery of T cell help, including ICOSL, CD40, and ICAM-1 on B cells, were normally expressed in SAP-deficient mice (Figures 3G–3I). T cells select B cells by providing them with signals that enhance their proliferation in the DZ (Gitlin et al., 2014, 2015; Victora et al., 2010). Analysis of proliferation by 5-ethynyl-2′-deoxyuridine (EdU) incorporation assay revealed no defect in B cell division within the GCs of SAP-deficient mice (Figure 3J). These results show that GC B cells in PPs have the capacity to receive help signals and proliferate independently of SAP-mediated T cell help.

SAP-Mediated T Cell Help Is Not Required for CSR to IgA and IgG2a/b

CSR to IgG1 isotype is highly dependent on the delivery of T cell help to B cells, whereas class switch to IgA can take place without T cells or CD40 signals (Al-Alem et al., 2005; Bergqvist et al., 2006, 2010; Macpherson et al., 2000). However, since GCs do not form in CD40- or TCRα-deficient mice, it remained unclear whether the formation of IgA^+ GC B cells depends on T cell functions. Our imaging experiments revealed that AID is expressed in the PPs of SAP-deficient mice, indicating that CSR can take place. To investigate the role of SAP in the formation of IgA^+ GC B cells, we compared the expression of IgM, IgG1, and IgA in GC cells derived from either WT or SAP-deficient mice by flow cytometric analysis. We found that IgG1 class switched B cells were nearly absent from the GCs of PPs, while the frequency of IgA^+ and IgM^+ GC B cells was increased (Figures 4A and 4B). Furthermore, IgA antibodies were detected in the intestinal contents of SAP-deficient mice, whereas no IgG1 antibodies were detected in the serum of these mice (Figures 4C and 4D). GC B cells in the PPs of SAP-deficient mice expressed AID (Figure 1C) in addition to transcripts of genes that mediate CSR, including *IL4Ra*, *IL21R*, *Stat3*, and *Stat6* (Figure 4E), suggesting that they are able to receive T cell help that induces CSR to IgG1. These experiments demonstrate that although the generation of IgA^+ GC B cells depends on T cell functions, it does not depend on SAP-mediated T cell help. Nonetheless, CSR to IgG1 is strictly SAP dependent, even when T cell-dependent GCs are generated. We conclude that T cell help to B cells, which promotes CSR to IgG1, is uncoupled from T cell functions that promote GC formation.

GCs were also detected in the GCs of SAP-deficient mice that were infected with influenza A virus (Figures 2G and 2H). These GCs contained primarily $IgG2a/b^+$ B cells, with very few $IgG1^+$ and IgA^+ B cells (Figure S4). As observed in the PPs of SAP-deficient mice, $IgG1^+$ B cells were absent from these GCs; however, the frequency of $IgG2a/b^+$ B cells was similar to those in WT mice (Figure S4). We conclude that CSR to IgG1 is strictly SAP dependent, whereas the generation of other isotypes does not depend on this molecule.

Clonal Diversification Is Uncoupled from Affinity-Dependent Selection Forces in PP GCs

The formation of GCs in SAP-deficient mice raises the question of whether these structures can support GC functions, including clonal diversification and selection of the best B cell variants for clonal expansion. To address this question, we sorted individual IgA^+ GL-7^+ FAS^+ B cells from single PPs of WT and SAP-deficient mice and sequenced their *Igh* mRNA, followed by CDR3-based clustering (Figure S5A). In WT mice, V-segment usage was highly diverse (~40/PP) and many clones bearing distinct CDR3s were detected within the GC compartment (Figures 5A and 5B). In contrast, in the PPs of SAP-deficient mice, we found limited V-segment usage (~15/PP), and analysis of the unique CDR3 sequence frequency revealed 2.17-fold fewer distinct B cell clones in the GCs of SAP^{KO} mice (Figures 5A and 5B). Furthermore, the GCs of SAP-deficient mice were dominated by 2–3 highly expanded clones, which consisted of 89.85% of the total GC cells (Figure 5B). To examine whether the reduced number of clones in the GCs of SAP-deficient mice is indeed a result of defects in T cell function in GCs, we depleted $CD4^+$ T cells by injecting anti-CD4 antibody into WT mice. After 2 weeks, although CD4 T cells were efficiently depleted within the PPs of WT mice, IgA^+ GC B cells were still evident (Figures S5B–S5D). Analysis of clonal diversity under these conditions

(B and C) Quantification of GC frequency in popliteal LNs (B) and PPs (C), as in (A).
(D) Representative flow cytometry plots and graph of dark zone (DZ) ($CXCR4^{hi}$ $CD86^{lo}$) and light zone (LZ) ($CXCR4^{lo}$ $CD86^{hi}$) GC B cell distribution in the PPs of WT and SAP^{KO} mice.
(E) Bcl6 transcript in sorted GC B cells derived from PPs of WT and SAP^{KO} mice.
(F) Representative histogram and quantification of Bcl6 expression in GC B cells of WT and SAP^{KO} mice; the naive B cell population is shown as a negative control.
(G) Representative images of mediastinal LNs of influenza-infected mice. LNs were fixed, sectioned, and stained for GL-7 (fluorescein isothiocyanate [FITC], green) and IgD (AF-647, white) to mark the GC and the B cell follicle. Hoechst was used for nuclear staining. Scale bar, 50 μm.
(H) Representative flow cytometry plots showing GC B cells ($Ki67^+$ $Bcl6^+$) in the mediastinal LNs of WT, SAP^{KO}, and $TCR\alpha^{KO}$ mice, 14 days following influenza infection. GC frequencies are summarized in the graph.
Data are pooled from two (B, D–F, and H) and four (C) independent experiments. Each dot represents a single mouse; line represents the mean. $^*p < 0.05$, $^{**}p < 0.01$, $^{***}p < 0.0001$, one-way ANOVA with Bonferroni posttest in (B), (C), and (H) and two-tailed Student's t test in (D)–(F). ns, not significant.

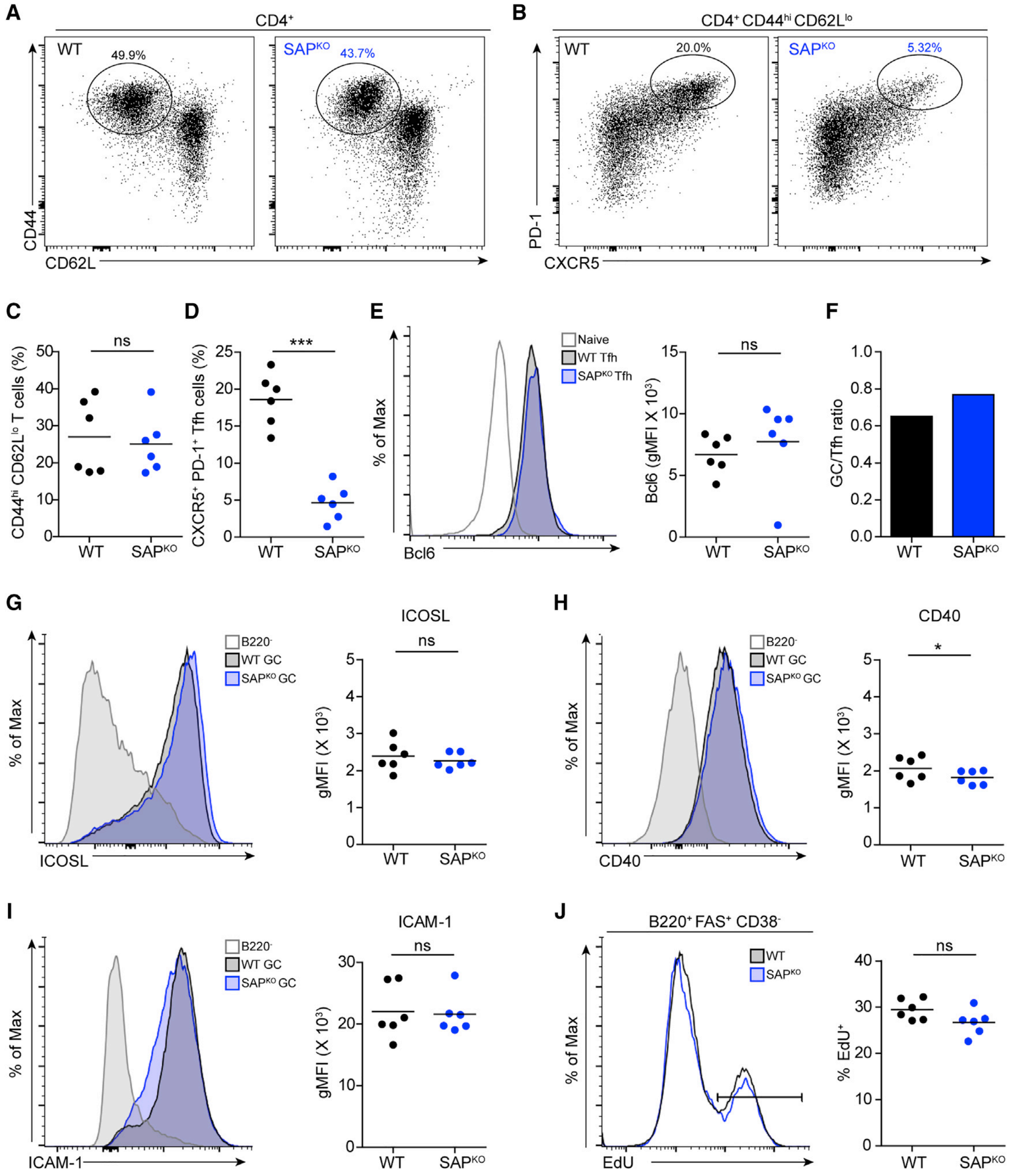

Figure 3. The Proportion of Tfh Cells in PP GCs Is Maintained in SAP-Deficient Mice

(A) Representative flow cytometry plots showing activated Th cell ($CD44^{hi}$ $CD62L^{lo}$) frequencies in the PPs of WT and SAP^{KO} mice.

(B) Representative flow cytometry plots showing Tfh cells ($CXCR5^+$ PD-1^+) gated from the activated T cell population in the PPs of WT and SAP^{KO} mice.

(C and D) Graphs showing the frequencies of $CD4^+$ activated (C) and Tfh cells (D), as in (A) and (B), respectively.

(E) Representative histogram showing Bcl6 expression in WT and SAP^{KO} Tfh cells. Expression in naive T cells is shown as a negative control, and frequencies are summarized in the graph.

(F) Graph showing the ratio between GC B cells and Tfh cells in WT and SAP^{KO} PPs.

(G–I) Representative histogram and quantification of ICOSL (G), CD40 (H), and ICAM-1 (I) expression in PP GC B cells.

(J) Representative flow cytometry plots showing EdU uptake by WT and SAP^{KO} GC B cells, 2.5 h following EdU administration. Frequencies of EdU^+ cells are summarized in the graph.

Data are pooled from two independent experiments, with three mice in each experiment. Each dot represents a single mouse; line represents the mean. *$p < 0.05$, ***$p < 0.0001$, two-tailed Student's t test. ns, not significant.

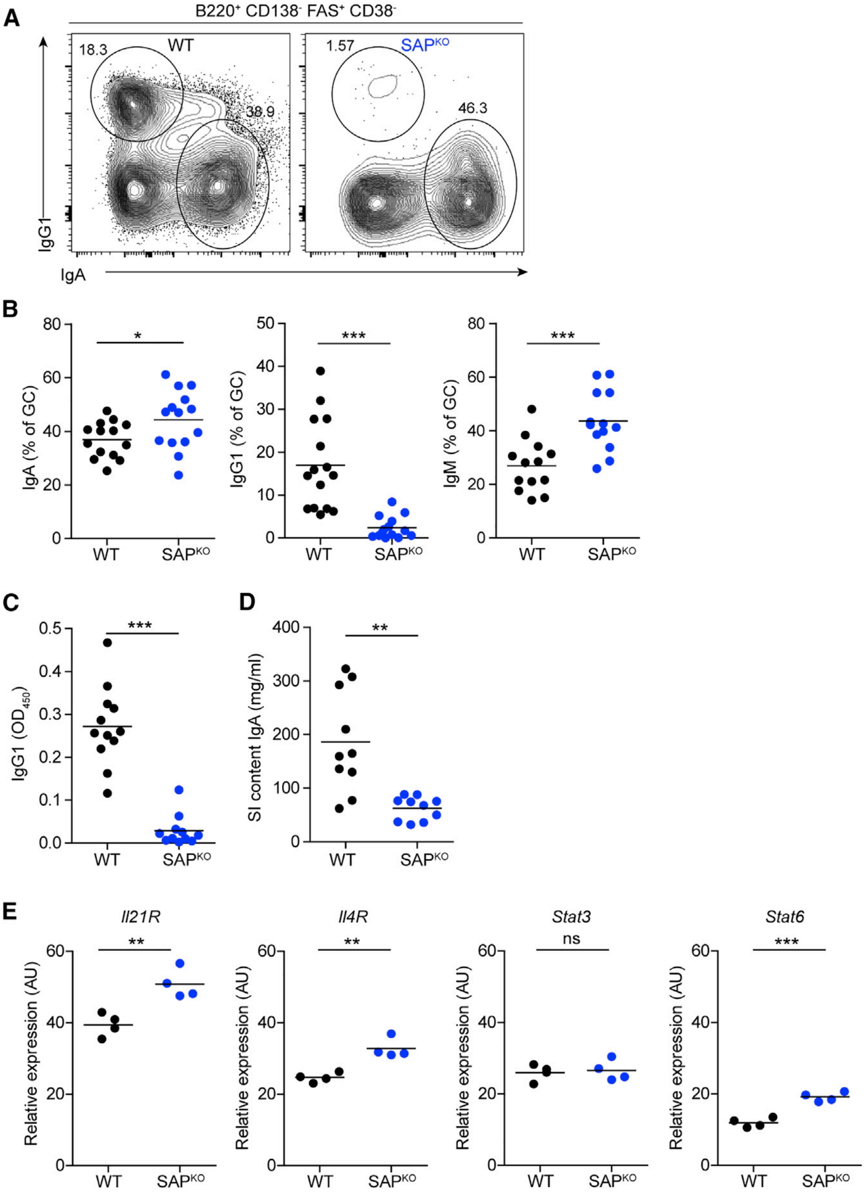

Figure 4. SAP-Mediated T Cell Help Is Not Required for Generation of IgA$^+$ GC B Cells in PPs

(A) Representative flow cytometry plots showing IgG1 and IgA isotype frequency gated from GC B cells derived from WT or SAP-deficient mice.

(B) Quantification of GC isotype frequencies, as shown in (A). Data are pooled from four independent experiments; each dot represents a single mouse; line represents the mean.

(C) Levels of IgG1 in the sera of WT and SAP^{KO} mice as estimated by standard ELISA.

(D) Quantification of IgA antibodies in the intestinal contents of WT and SAP^{KO} mice as measured by ELISA.

(E) qPCR for IL21R, IL4R, STAT3, and STAT6 transcripts in sorted GC B cells (GL7$^+$ FAS$^+$) derived from PPs of WT and SAP^{KO} mice.

Data are pooled from two independent experiments with two mice in each experiment. Each dot represents a single mouse, line represents the mean. *p < 0.05, **p < 0.01, ***p < 0.0001, two-tailed Student's t test. ns, not significant.

revealed a 2-fold reduction in V-segment usage and a 2.2-fold decrease in the frequency of individual CDR3 sequences (Figures 5A and 5B). We conclude that SAP-mediated T cell functions promote clonal diversity in the GC reaction within PPs.

GC B cells express AID, an enzyme that mediates random SHM and diversification of clones over time, followed by selection of the high-affinity variants. Since GC B cells in SAP-deficient mice express AID, we examined whether SAP is required for clonal diversification by SHM followed by a selection stage. Comparison of the mutation number per cell revealed that GC B cells in SAP-deficient mice accumulated a significant number of mutations in their immunoglobulins, although to a lesser extent than in WT mice (Figure 5C). Although clonal diversity was reduced in anti-CD4 treated mice (Figures 5A and 5B), reduction in the number of SHM per cell was not observed, suggesting that these highly mutated cells persisted after CD4 depletion within PP GCs in a T cell-independent manner (Figure 5C). These findings indicate that SHM and clonal diversification within the GC reaction can take place in the absence of SAP-mediated T cell help.

To examine whether positive selection takes place in the GCs of SAP-deficient mice, we reconstructed lineage trees of clones recovered from PPs. In WT mice, we found several families of clones that exhibited clonal diversification by progressive SHM accumulation and by clonal bursts (Figure 5D). Analysis of the dominant clones in SAP-deficient mice revealed extensive clonal diversification, including clonal burst events that gave rise to many diverse progeny B cells (Figure 5E). These clonal lineages contained more members compared to WT GC B cells, since more clonally related cells were recovered from SAP-deficient mice, as a result of lower clonal diversity in these GCs (Figures 5A and 5B). Similar results were observed in anti-CD4 treated mice (Figure 5F). Thus, progressive accumulation of SHM and clonal bursts can take place in GC B cells within PPs in a SAP-independent manner.

To investigate whether B cell selection forces play a role in the GCs of SAP-deficient mice, we applied BASELINe analysis to quantify the ratio of synonymous to non-synonymous nucleotide changes compared to null model expectations in the framework (FWR) and complementary determining regions (CDRs) of the *Igh* sequences (Cui et al., 2016; Yaari et al., 2012). Synonymous (silent) mutations do not change the encoded amino acid, and thus do not contribute to antibody affinity maturation,

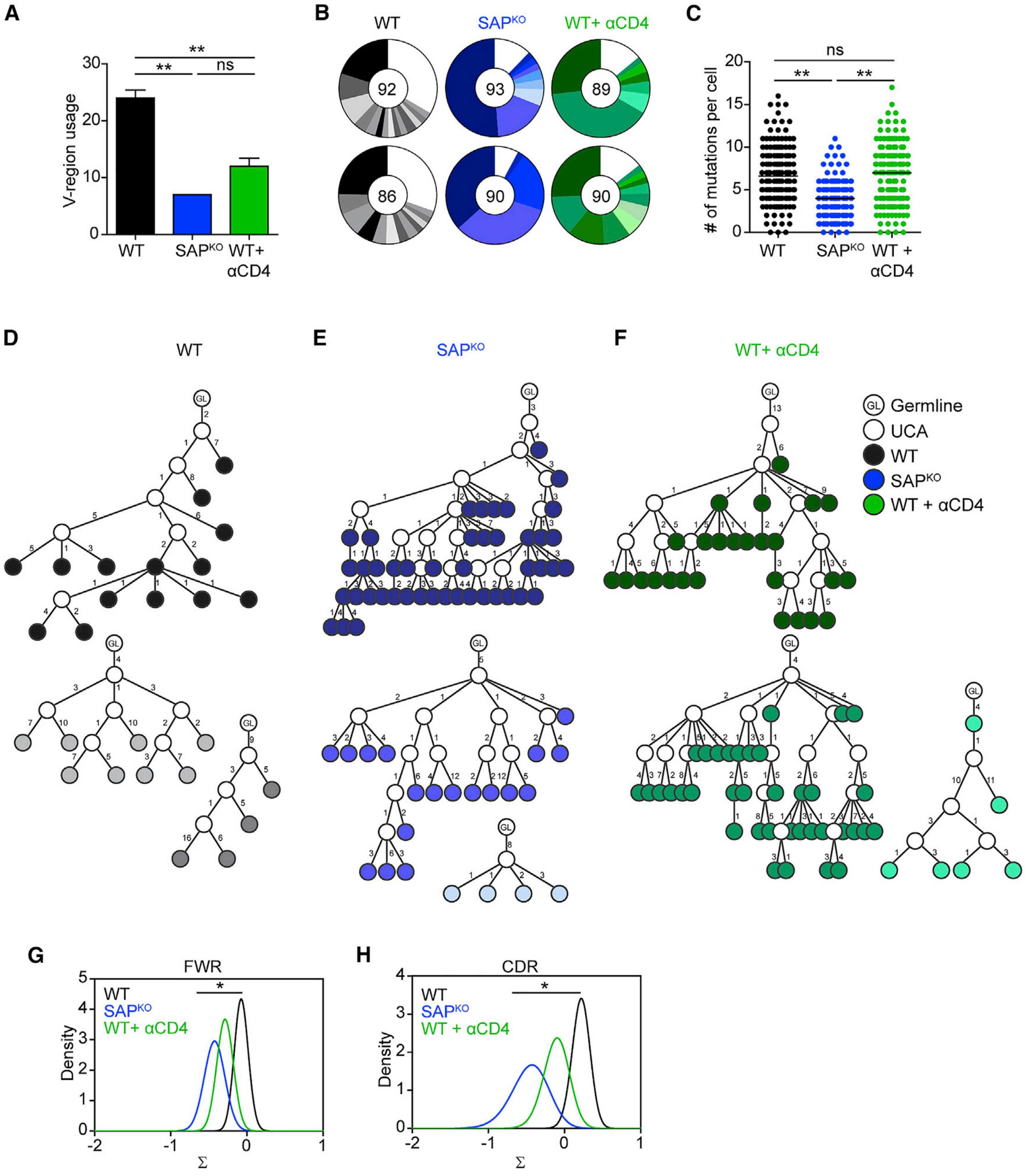

Figure 5. Clonal Diversification Is Uncoupled from Selection Forces in PP GCs

(A) The number of V-regions detected in IgA sequences of GC-derived ($GL7^+$ FAS^+ IgA^+) B cells recovered from a single PP derived from either WT, SAP^{KO}, or WT mice treated with αCD4-depleting antibody for 14 days (WT + αCD4).

(B) Clonal distribution based on CDR3 sequences, as in (A). Colored fractions represent expanded CDR3 sequences (>2); white fraction represents single clones. Each pie chart represents one mouse (n = 2). The number of sequenced cells is indicated in the center circle.

(C) Number of mutations per B cell of the cells described in (A). Data are pooled from two independent experiments with one mouse in each experiment. **$p < 0.01$, one way ANOVA with Bonferroni posttest. ns, not significant.

(D–F) Lineage-tree analysis of clonally related sequences in WT (D), SAP^{KO} (E), and WT + αCD4 (F) GC B cells. The number of mutations between neighboring nodes is indicated and includes synonymous, non-synonymous, and reverse mutations to the germline sequence. GL, germline; UCA, unique common ancestor, inferred from the sequence analysis.

(G and H) Graphs showing the density of selection strength for all analyzed sequences within the framework (FWR) (G) or complementary determining region (CDR) (H) of WT, SAP^{KO}, and αCD4 treated mice. Selection was estimated using BASELINe focused test with the RS5NF mutability model. *False discovery rate (FDR) < 0.05.

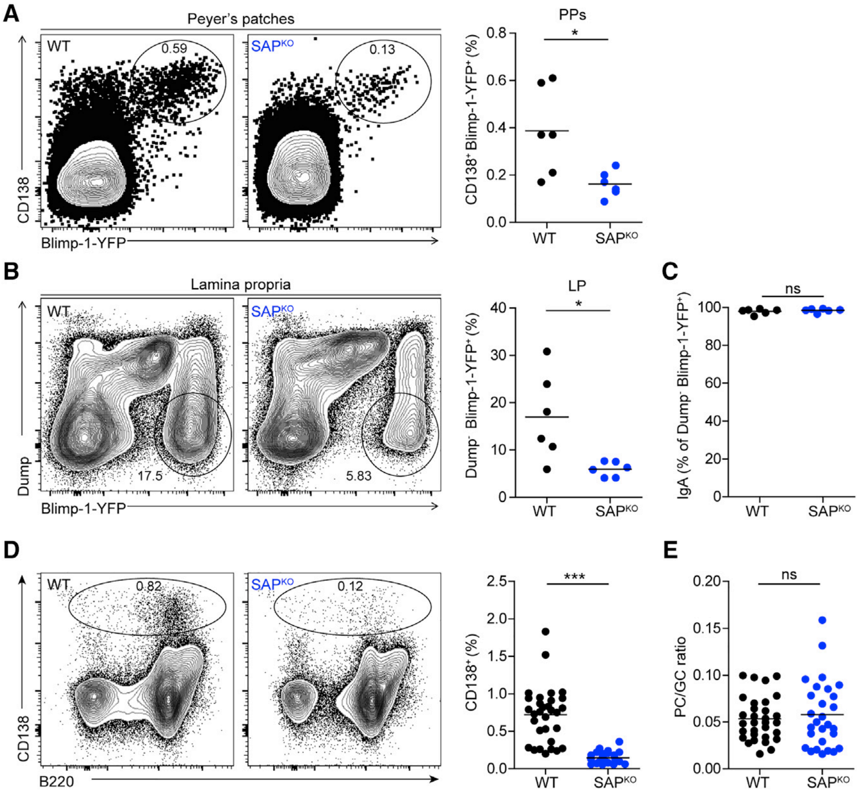

Figure 6. PC Generation in PPs Is Regulated by SAP in an Indirect Manner

(A) Representative flow cytometry plots and quantification of Blimp-1^{+} CD138^{+} cell population in the PPs of WT and SAP^{KO} Blimp-1-YFP reporter mice.

(B) Representative flow cytometry plots and quantification of the Blimp-1^{+} Dump^{-} (CD4^{+} CD8^{+} F4/80^{+} Gr-1^{+}) cell population in the lamina propria of WT and SAP^{KO} Blimp-1-YFP reporter mice.

(C) Surface IgA frequency gated from the lamina propria Blimp-1^{+} cells shown in (B).

(D) Representative flow cytometry plots and quantification of CD138^{+} cell population in PPs of WT and SAP^{KO} mice.

(E) Graph showing the ratio between the frequency of CD138^{+} cells to GC size in PPs of WT and SAP-deficient mice.

In (A)–(C), data are pooled from two independent experiments with three mice in each experiment; line represents the mean. In (D) and (E), data are pooled from five independent experiments, with coupled CD138 and GC staining; line represents the mean. *$p < 0.05$, ***$p < 0.0001$, two-tailed Student's t test. ns, not significant.

whereas non-synonymous mutations in the DNA, which result in changes in the nucleic acid code, may affect Ig affinity (Yaari et al., 2012). Hence, affinity-based clonal selection is characterized by an increase in the ratio of replacement versus silent mutations. We found that B cells in SAP-deficient mice showed a lower ratio of synonymous to non-synonymous mutations, both in the CDR and FWR sequences, compared to WT mice (Figures 5G and 5H). Furthermore, mice that were treated with anti-CD4 antibody for 2 weeks also showed a reduction in selection strength, but to a lesser extent than that observed in SAP-deficient mice (Figures 5G and 5H). Similar differences were observed when clones bearing the same V genes were compared between WT and SAP-deficient mice (Figure S6). Since the BASELINe analysis compares each Ig sequence to its corresponding germline sequence, independent of clonal expansion, the difference in clonal divergence did not affect the results of this analysis. We conclude that selection forces within chronic GCs of the PP depend on SAP, whereas clonal diversification can take place in the absence of SAP-dependent T cell help.

Positive selection of B cells by T cells in GCs depends on the upregulation of Myc and its downstream genes (Calado et al., 2012; Dominguez-Sola et al., 2012). To understand why positive selection is perturbed in the absence of SAP in PPs, we sorted GC B cells from WT and SAP-deficient mice and examined their transcriptome by bulk RNA sequencing analysis. We found that GC B cells derived from SAP-deficient mice show altered gene expression profiles (Figure S5F). Gene set enrichment analysis (GSEA) revealed a reduction in Myc targets gene expression (Mootha et al., 2003; Subramanian et al., 2005). Thus, in the absence of SAP, GC B cells are unable to respond efficiently to T cell help and upregulate Myc-responsive genes that promote their selection and provide a differential advantage in clonal expansion (Figure S5G).

Plasma Cell Generation in PPs Is Regulated by SAP in an Indirect Manner

T cell help to B cells is essential for the effective differentiation of GC B cells into PCs. In intestinal tissues, most of the IgA^{+} plasma cells are mutated in their *Igh* variable regions, indicating that they originate from PP GCs (Lindner et al., 2012). To examine whether SAP regulates the formation of PCs in PPs and the gut lamina propria, we generated SAP-deficient mice that express yellow fluorescent protein (YFP) under the promoter of the PC transcription factor Blimp-1 (Blimp-1-YFP). Flow cytometric analysis of cells derived from the PP and lamina propria of Blimp-1-YFP reporter mice revealed 2.39-fold and 2.86-fold, respectively, fewer Blimp-1^{+} cells in SAP-deficient mice compared to WT mice (Figures 6A and 6B). Furthermore, nearly all of the Blimp-1-YFP^{+} cells in the lamina propria of both WT and SAP^{KO} mice were IgA^{+} plasma cells (Figure 6C). These findings suggest that SAP is required for PC formation; however, the diminished number of Blimp-1^{+} cells may also be a result of the small size of GCs formed in SAP-deficient mice. To examine this possibility, we stained PP cells derived from SAP-deficient mice for the PC marker CD138 and normalized the cell frequency to the GC size. Flow cytometric analysis revealed that the PPs of SAP-deficient mice contain 5-fold fewer plasma cells compared to WT mice (Figure 6D). However, the PC:GC cell ratio was similar between WT and SAP-deficient mice (Figure 6E). Thus, we

conclude that SAP controls PC formation in the gut at least partially through regulation of the GC size.

DISCUSSION

Tfh cells are essential for GC formation and function in inducible GCs in response to immunization and pathogen invasion (Vinuesa et al., 2010). Here, we defined the role of SAP-mediated T cell functions in chronic GCs within PPs. We demonstrate the following: (1) in PPs, small bona fide chronic GCs are formed in the absence of SAP-dependent T cell help that host IgA^+ B cells and generate PCs; (2) B cell diversification in these GCs through progressive SHM and clonal bursts is SAP independent; and (3) T cell-imposed selection forces in PP GCs require SAP. Thus, as opposed to B cell responses to model antigens and some viral infections in peripheral LNs, we find that in chronic GCs within PPs, SAP is not essential for most T cell-dependent B cell functions.

SAP is essential for GC formation in response to immunization (Schwartzberg et al., 2009), and it is most likely that its functional dichotomy between the LNs and PPs can be attributed to the chronic nature of the PP GC reaction and the harsh environment of the gut. Constant exposure to antigens may provide sufficient stimulation over time to support the formation of Tfh cells and GC seeding in the PPs, even when T cell functions are defective. It was previously demonstrated that Tfh cell development and functions in the LNs of SAP-deficient mice can be recovered upon injection of a large amount of cognate peptide to mice (Deenick et al., 2010). This model may represent a chronic antigen exposure scenario or a response to a large number of T cell antigens. It was shown that SAP-independent GC seeding does not take place in the spleen, even during chronic viral infection; however, close examination of the data suggests that a very small GC population does form in the infected mice (Crotty et al., 2006). We repeated these experiments and identified a small but significant GC cell population in mice that were infected with chronic $LCMV_{cl13}$, which persists for a long time in the mouse. Furthermore, it was found that in chronic *Plasmodium* infection of SAP-deficient mice, small GCs were detected several weeks after exposure to the parasite, although GC functions such as SHM and affinity maturation were not examined (Pérez-Mazliah et al., 2017). In addition, it was demonstrated that GCs are not formed in SAP-deficient mice that were infected with influenza virus (Kamperschroer et al., 2006); however, we found here that B cells in SAP-deficient mice that received a different dose of a different strain of influenza virus were able to form GCs. This observation is consistent with previous findings that show the generation of virus-specific class-switched antibodies in the lungs, independent of MHC class II expression on B cells (Sangster et al., 2003). These findings suggest that antigen-specific responses in GCs within the lungs can occur to some extent without cognate T cell help (Denton et al., 2019; Lee et al., 2005; Sangster et al., 2003). Thus, it seems that long-lasting chronic exposure to antigens can drive GC formation, even in the absence of SAP-dependent T cell help.

An additional mechanism that may bypass the need for SAP could be danger signals derived from gut-resident bacteria, independent of specific antigen recognition (Slack et al., 2014). Although we did not exclude this possibility, this scenario is unlikely since non-specific B cell activation would lead to the expansion of polyclonal B cells, independent of antigen recognition, whereas GCs in SAP-deficient mice host few clones; nonetheless, the combination of microbial-derived signals with BCR ligation may trigger the GC response in SAP-deficient mice.

Since SAP-dependent T cell signals are not essential for GC formation in PPs, this type of T cell help may support a response against non-protein antigens. In adult mice and humans, nearly all of the IgA sequences recovered from lamina propria resident B cells were found to be highly mutated, suggesting that B cells that carry a BCR that is specific to a non-protein antigen can acquire SHM (Lindner et al., 2015). Highly mutated and expanded LPS-specific B cells were detected in the guts of both humans and mice (Kauffman et al., 2016; Lindner et al., 2015). Thus, it is possible that upon antigen encounter, naive or memory B cells can invade pre-existing GCs, wherein cognate T cell help is not a prerequisite for participating in the GC reaction. The mechanism of clonal diversification without cognate Tfh cells in GCs may efficiently expand the circulating memory B cell pool, and thereby continually fuel the clonally stable PC population in the lamina propria and support a progressive increase or a burst in SHM (Bemark et al., 2016; Bergqvist et al., 2013; Lindner et al., 2015). In addition, this pathway can contribute to the generation of low-affinity B cell clones within the chronic GCs of the PP and therefore support the mutualism between the host and microbiota (Gutzeit et al., 2014; Slack et al., 2014). In addition, we found that the ratio of Tfh cells:GC B cells was intact in PPs, indicating that sufficient numbers of Tfh cells were formed in SAP-deficient mice. However, we could not determine whether these cells are specific to the same antigen presented by GC B cells in the PPs. Regardless of antigen specificity in the chronic GCs that are formed in SAP-deficient mice, our findings demonstrate that clonal diversification can take place without the involvement of SAP-dependent T cell help. Nonetheless, competition for T cell help and clonal selection depend on SAP expression and antigen presentation (Biram et al., 2019a). These findings are reminiscent of the events that occur in the SED, wherein clonal expansion and PC formation take place in a T cell-dependent manner; however, selection of the high-affinity clones by T cells does not take place at this site (Biram et al., 2019a).

Collectively, our findings suggest that Tfh cells in the gut function differently than in other organs following an immunization or viral infection, indicating that SAP-dependent and SAP-independent GC reactions co-exist in PPs. These observations suggest that the traditional classification of the B cell immune responses as T cell-dependent or T cell-independent responses does not describe the full complexity of the GC reaction and, in particular, chronic GCs, as in PPs. Understanding immune-cell interactions in chronic GCs under homeostatic conditions may open new avenues in the development of oral vaccination strategies.

STAR★METHODS

Detailed methods are provided in the online version of this paper and include the following:

- KEY RESOURCES TABLE
- LEAD CONTACT AND MATERIALS AVAILABILITY

- EXPERIMENTAL MODEL AND SUBJECT DETAILS
  - Mice
  - Immunizations, treatments and viral infections
- METHOD DETAILS
  - ELISA
  - *In vivo* EdU proliferation assay
  - Flow cytometry
  - Quantitative PCR analysis
  - Immunohistochemistry
  - Image acquisition by TPLSM
  - Image analysis
  - Single cell Igh sequencing
  - Igh sequence analysis
  - RNA sequencing and gene set enrichment analysis
- QUANTIFICATION AND STATISTICAL ANALYSIS
- DATA AND CODE AVAILABILITY

SUPPLEMENTAL INFORMATION

Supplemental Information can be found online at https://doi.org/10.1016/j.celrep.2020.01.032.

ACKNOWLEDGMENTS

Z.S. is supported by European Research Council (ERC) grant no. 677713, the Human Frontiers of Science Program (CDA-00023/2016), Israel Science Foundation (ISF) grant no. 1090/18, the Azrieli Foundation, the Rising Tide Foundation, and the Morris Kahn Institute for Human Immunology. Z.S. and M.A.L. are members of the European Molecular Biology Organization (EMBO) Young Investigator Program. Z.S. is supported by grants from the Benoziyo Endowment Fund for the Advancement of Science, The Sir Charles Clore Research Prize, the Comisaroff Family Trust, the Irma & Jacques Ber-Lehmsdorf Foundation, the Gerald O. Mann Charitable Foundation, and the David M. Polen Charitable Trust. G.Y. is supported by Israel Science Foundation (ISF) grant no. 832/16. M.A.L. is supported by the Biotechnology and Biological Sciences Research Council (BBS/E/B/000C0407 and BBS/E/B/000C0427). A.E.D. is supported by a Biotechnology and Biological Sciences Research Council Future Leader Fellowship (BB/N011740/1).

AUTHOR CONTRIBUTIONS

Conceptualization, A.B. and Z.S.; Formal Analysis, A.B. and E.W.; Investigation, A.B., A.E.D., and I.Z.; Resources, M.A.L.; Data Curation, E.W. and G.Y.; Writing – Original Draft, A.B. and Z.S.; Writing – Review & Editing, A.B. and Z.S.; Visualization, A.B.; Funding Acquisition, Z.S.; Supervision, Z.S., G.Y., M.A.L., and M.B.

DECLARATION OF INTERESTS

The authors declare no competing interests.

Received: June 12, 2019
Revised: October 24, 2019
Accepted: January 8, 2020
Published: February 11, 2020

REFERENCES

Al-Alem, U., Li, C., Forey, N., Relouzat, F., Fondanèche, M.C., Tavtigian, S.V., Wang, Z.Q., Latour, S., and Yin, L. (2005). Impaired Ig class switch in mice deficient for the X-linked lymphoproliferative disease gene Sap. Blood *106*, 2069–2075.

Anders, S., Pyl, P.T., and Huber, W. (2015). HTSeq–a Python framework to work with high-throughput sequencing data. Bioinformatics *31*, 166–169.

Basso, K., and Dalla-Favera, R. (2012). Roles of BCL6 in normal and transformed germinal center B cells. Immunol. Rev. *247*, 172–183.

Bemark, M., Sale, J.E., Kim, H.-J., Berek, C., Cosgrove, R.A., and Neuberger, M.S. (2000). Somatic hypermutation in the absence of DNA-dependent protein kinase catalytic subunit (DNA-PK$_{(cs)}$) or recombination-activating gene (RAG)1 activity. J. Exp. Med. *192*, 1509–1514.

Bemark, M., Hazanov, H., Strömberg, A., Komban, R., Holmqvist, J., Köster, S., Mattsson, J., Sikora, P., Mehr, R., and Lycke, N.Y. (2016). Limited clonal relatedness between gut IgA plasma cells and memory B cells after oral immunization. Nat. Commun. *7*, 12698.

Berek, C., Berger, A., and Apel, M. (1991). Maturation of the immune response in germinal centers. Cell *67*, 1121–1129.

Bergqvist, P., Gärdby, E., Stensson, A., Bemark, M., and Lycke, N.Y. (2006). Gut IgA class switch recombination in the absence of CD40 does not occur in the lamina propria and is independent of germinal centers. J. Immunol. *177*, 7772–7783.

Bergqvist, P., Stensson, A., Lycke, N.Y., and Bemark, M. (2010). T cell-independent IgA class switch recombination is restricted to the GALT and occurs prior to manifest germinal center formation. J. Immunol. *184*, 3545–3553.

Bergqvist, P., Stensson, A., Hazanov, L., Holmberg, A., Mattsson, J., Mehr, R., Bemark, M., and Lycke, N.Y. (2013). Re-utilization of germinal centers in multiple Peyer's patches results in highly synchronized, oligoclonal, and affinity-matured gut IgA responses. Mucosal Immunol. *6*, 122–135.

Biram, A., Strömberg, A., Winter, E., Stoler-Barak, L., Salomon, R., Addadi, Y., Dahan, R., Yaari, G., Bemark, M., and Shulman, Z. (2019a). BCR affinity differentially regulates colonization of the subepithelial dome and infiltration into germinal centers within Peyer's patches. Nat. Immunol. *20*, 482–492.

Biram, A., Davidzohn, N., and Shulman, Z. (2019b). T cell interactions with B cells during germinal center formation, a three-step model. Immunol. Rev. *288*, 37–48.

Calado, D.P., Sasaki, Y., Godinho, S.A., Pellerin, A., Köchert, K., Sleckman, B.P., de Alborán, I.M., Janz, M., Rodig, S., and Rajewsky, K. (2012). The cell-cycle regulator c-Myc is essential for the formation and maintenance of germinal centers. Nat. Immunol. *13*, 1092–1100.

Cannons, J.L., Yu, L.J., Jankovic, D., Crotty, S., Horai, R., Kirby, M., Anderson, S., Cheever, A.W., Sher, A., and Schwartzberg, P.L. (2006). SAP regulates T cell-mediated help for humoral immunity by a mechanism distinct from cytokine regulation. J. Exp. Med. *203*, 1551–1565.

Cannons, J.L., Qi, H., Lu, K.T., Dutta, M., Gomez-Rodriguez, J., Cheng, J., Wakeland, E.K., Germain, R.N., and Schwartzberg, P.L. (2010). Optimal germinal center responses require a multistage T cell:B cell adhesion process involving integrins, SLAM-associated protein, and CD84. Immunity *32*, 253–265.

Casola, S., Otipoby, K.L., Alimzhanov, M., Humme, S., Uyttersprot, N., Kutok, J.L., Carroll, M.C., and Rajewsky, K. (2004). B cell receptor signal strength determines B cell fate. Nat. Immunol. *5*, 317–327.

Chu, C., Wang, Y., Zhang, X., Ni, X., Cao, J., Xu, W., Dong, Z., Yuan, P., Wei, W., Ma, Y., et al. (2014). SAP-regulated T Cell-APC adhesion and ligation-dependent and -independent Ly108-CD3ζ interactions. J. Immunol. *193*, 3860–3871.

Corcoran, L.M., and Tarlinton, D.M. (2016). Regulation of germinal center responses, memory B cells and plasma cell formation-an update. Curr. Opin. Immunol. *39*, 59–67.

Craig, S.W., and Cebra, J.J. (1971). Peyer's patches: an enriched source of precursors for IgA-producing immunocytes in the rabbit. J. Exp. Med. *134*, 188–200.

Crotty, S., Kersh, E.N., Cannons, J., Schwartzberg, P.L., and Ahmed, R. (2003). SAP is required for generating long-term humoral immunity. Nature *421*, 282–287.

Crotty, S., McCausland, M.M., Aubert, R.D., Wherry, E.J., and Ahmed, R. (2006). Hypogammaglobulinemia and exacerbated CD8 T-cell-mediated immunopathology in SAP-deficient mice with chronic LCMV infection mimics human XLP disease. Blood *108*, 3085–3093.

Cui, A., Di Niro, R., Vander Heiden, J.A., Briggs, A.W., Adams, K., Gilbert, T., O'Connor, K.C., Vigneault, F., Shlomchik, M.J., and Kleinstein, S.H. (2016). A Model of Somatic Hypermutation Targeting in Mice Based on High-Throughput Ig Sequencing Data. J. Immunol. *197*, 3566–3574.

Deenick, E.K., Chan, A., Ma, C.S., Gatto, D., Schwartzberg, P.L., Brink, R., and Tangye, S.G. (2010). Follicular helper T cell differentiation requires continuous antigen presentation that is independent of unique B cell signaling. Immunity *33*, 241–253.

Denton, A.E., Innocentin, S., Carr, E.J., Bradford, B.M., Lafouresse, F., Mabbott, N.A., Mörbe, U., Ludewig, B., Groom, J.R., Good-Jacobson, K.L., and Linterman, M.A. (2019). Type I interferon induces CXCL13 to support ectopic germinal center formation. J. Exp. Med. *216*, 621–637.

Dobin, A., Davis, C.A., Schlesinger, F., Drenkow, J., Zaleski, C., Jha, S., Batut, P., Chaisson, M., and Gingeras, T.R. (2013). STAR: ultrafast universal RNA-seq aligner. Bioinformatics *29*, 15–21.

Dominguez-Sola, D., Victora, G.D., Ying, C.Y., Phan, R.T., Saito, M., Nussenzweig, M.C., and Dalla-Favera, R. (2012). The proto-oncogene MYC is required for selection in the germinal center and cyclic reentry. Nat. Immunol. *13*, 1083–1091.

Eisen, H.N., and Siskind, G.W. (1964). Variations in affinities of antibodies during the immune response. Biochemistry *3*, 996–1008.

Felsenstein, J. (2005). PHYLIP (Phylogeny Inference Package) version 3.6 (Department of Genome Sciences, University of Washington).

Gibbons, D.L., and Spencer, J. (2011). Mouse and human intestinal immunity: same ballpark, different players; different rules, same score. Mucosal Immunol. *4*, 148–157.

Gitlin, A.D., Shulman, Z., and Nussenzweig, M.C. (2014). Clonal selection in the germinal centre by regulated proliferation and hypermutation. Nature *509*, 637–640.

Gitlin, A.D., Mayer, C.T., Oliveira, T.Y., Shulman, Z., Jones, M.J.K., Koren, A., and Nussenzweig, M.C. (2015). HUMORAL IMMUNITY. T cell help controls the speed of the cell cycle in germinal center B cells. Science *349*, 643–646.

Gupta, N.T., Vander Heiden, J.A., Uduman, M., Gadala-Maria, D., Yaari, G., and Kleinstein, S.H. (2015). Change-O: a toolkit for analyzing large-scale B cell immunoglobulin repertoire sequencing data. Bioinformatics *31*, 3356–3358.

Gutzeit, C., Magri, G., and Cerutti, A. (2014). Intestinal IgA production and its role in host-microbe interaction. Immunol. Rev. *260*, 76–85.

Hapfelmeier, S., Lawson, M.A.E., Slack, E., Kirundi, J.K., Stoel, M., Heikenwalder, M., Cahenzli, J., Velykoredko, Y., Balmer, M.L., Endt, K., et al. (2010). Reversible microbial colonization of germ-free mice reveals the dynamics of IgA immune responses. Science *328*, 1705–1709.

Ho, I.Y., Bunker, J.J., Erickson, S.A., Neu, K.E., Huang, M., Cortese, M., Pulendran, B., and Wilson, P.C. (2016). Refined protocol for generating monoclonal antibodies from single human and murine B cells. J. Immunol. Methods *438*, 67–70.

Jaitin, D.A., Kenigsberg, E., Keren-Shaul, H., Elefant, N., Paul, F., Zaretsky, I., Mildner, A., Cohen, N., Jung, S., Tanay, A., and Amit, I. (2014). Massively parallel single-cell RNA-seq for marker-free decomposition of tissues into cell types. Science *343*, 776–779.

Jones, L., Ho, W.Q., Ying, S., Ramakrishna, L., Srinivasan, K.G., Yurieva, M., Ng, W.P., Subramaniam, S., Hamadee, N.H., Joseph, S., et al. (2016). A subpopulation of high IL-21-producing CD4(+) T cells in Peyer's Patches is induced by the microbiota and regulates germinal centers. Sci. Rep. *6*, 30784.

Kageyama, R., Cannons, J.L., Zhao, F., Yusuf, I., Lao, C., Locci, M., Schwartzberg, P.L., and Crotty, S. (2012). The receptor Ly108 functions as a SAP adaptor-dependent on-off switch for T cell help to B cells and NKT cell development. Immunity *36*, 986–1002.

Kamperschroer, C., Dibble, J.P., Meents, D.L., Schwartzberg, P.L., and Swain, S.L. (2006). SAP is required for Th cell function and for immunity to influenza. J. Immunol. *177*, 5317–5327.

Kauffman, R.C., Bhuiyan, T.R., Nakajima, R., Mayo-Smith, L.M., Rashu, R., Hoq, M.R., Chowdhury, F., Khan, A.I., Rahman, A., Bhaumik, S.K., et al. (2016). Single-cell analysis of the plasmablast response to Vibrio cholerae demonstrates expansion of cross-reactive memory B cells. MBio *7*, e02021-16.

Kohen, R., Barlev, J., Hornung, G., Stelzer, G., Feldmesser, E., Kogan, K., Safran, M., and Leshkowitz, D. (2019). UTAP: User-friendly Transcriptome Analysis Pipeline. BMC Bioinformatics *20*, 154.

Kometani, K., and Kurosaki, T. (2015). Differentiation and maintenance of long-lived plasma cells. Curr. Opin. Immunol. *33*, 64–69.

Lee, B.O., Rangel-Moreno, J., Moyron-Quiroz, J.E., Hartson, L., Makris, M., Sprague, F., Lund, F.E., and Randall, T.D. (2005). CD4 T cell-independent antibody response promotes resolution of primary influenza infection and helps to prevent reinfection. J. Immunol. *175*, 5827–5838.

Lindner, C., Wahl, B., Föhse, L., Suerbaum, S., Macpherson, A.J., Prinz, I., and Pabst, O. (2012). Age, microbiota, and T cells shape diverse individual IgA repertoires in the intestine. J. Exp. Med. *209*, 365–377.

Lindner, C., Thomsen, I., Wahl, B., Ugur, M., Sethi, M.K., Friedrichsen, M., Smoczek, A., Ott, S., Baumann, U., Suerbaum, S., et al. (2015). Diversification of memory B cells drives the continuous adaptation of secretory antibodies to gut microbiota. Nat. Immunol. *16*, 880–888.

Liu, D., Xu, H., Shih, C., Wan, Z., Ma, X., Ma, W., Luo, D., and Qi, H. (2015). T-B-cell entanglement and ICOSL-driven feed-forward regulation of germinal centre reaction. Nature *517*, 214–218.

Love, M.I., Huber, W., and Anders, S. (2014). Moderated estimation of fold change and dispersion for RNA-seq data with DESeq2. Genome Biol. *15*, 550.

Macpherson, A.J., Gatto, D., Sainsbury, E., Harriman, G.R., Hengartner, H., and Zinkernagel, R.M. (2000). A primitive T cell-independent mechanism of intestinal mucosal IgA responses to commensal bacteria. Science *288*, 2222–2226.

Martin, M. (2011). Cutadapt removes adapter sequences from high-throughput sequencing reads. EMBnet J. *17*, 10.

McCausland, M.M., Yusuf, I., Tran, H., Ono, N., Yanagi, Y., and Crotty, S. (2007). SAP regulation of follicular helper CD4 T cell development and humoral immunity is independent of SLAM and Fyn kinase. J. Immunol. *178*, 817–828.

Mombaerts, P., Mizoguchi, E., Ljunggren, H.G., Iacomini, J., Ishikawa, H., Wang, L., Grusby, M.J., Glimcher, L.H., Winn, H.J., Bhan, A.K., et al. (1994). Peripheral lymphoid development and function in TCR mutant mice. Int. Immunol. *6*, 1061–1070.

Mootha, V.K., Lindgren, C.M., Eriksson, K.-F., Subramanian, A., Sihag, S., Lehar, J., Puigserver, P., Carlsson, E., Ridderstråle, M., Laurila, E., et al. (2003). PGC-1α-responsive genes involved in oxidative phosphorylation are coordinately downregulated in human diabetes. Nat. Genet. *34*, 267–273.

Pérez-Mazliah, D., Nguyen, M.P., Hosking, C., McLaughlin, S., Lewis, M.D., Tumwine, I., Levy, P., and Langhorne, J. (2017). Follicular Helper T Cells are Essential for the Elimination of Plasmodium Infection. EBioMedicine *24*, 216–230.

Qi, H., Cannons, J.L., Klauschen, F., Schwartzberg, P.L., and Germain, R.N. (2008). SAP-controlled T-B cell interactions underlie germinal centre formation. Nature *455*, 764–769.

Reboldi, A., and Cyster, J.G. (2016). Peyer's patches: organizing B-cell responses at the intestinal frontier. Immunol. Rev. *271*, 230–245.

Reboldi, A., Arnon, T.I., Rodda, L.B., Atakilit, A., Sheppard, D., and Cyster, J.G. (2016). IgA production requires B cell interaction with subepithelial dendritic cells in Peyer's patches. Science *352*, aaf4822.

Rommel, P.C., Bosque, D., Gitlin, A.D., Croft, G.F., Heintz, N., Casellas, R., Nussenzweig, M.C., Kriaucionis, S., and Robbiani, D.F. (2013). Fate mapping for activation-induced cytidine deaminase (AID) marks non-lymphoid cells during mouse development. PLoS One *8*, e69208.

Sammicheli, S., Kuka, M., Di Lucia, P., Jimenez de Oya, N.J., De Giovanni, M., Fioravanti, J., Cristofani, C., Maganuco, C.G., Fallet, B., Ganzer, L., et al. (2016). Inflammatory monocytes hinder antiviral B cell responses. Sci. Immunol. *1*, eaah6789.

Sangster, M.Y., Riberdy, J.M., Gonzalez, M., Topham, D.J., Baumgarth, N., and Doherty, P.C. (2003). An early CD4+ T cell-dependent immunoglobulin A

response to influenza infection in the absence of key cognate T-B interactions. J. Exp. Med. *198*, 1011–1021.

Schwartzberg, P.L., Mueller, K.L., Qi, H., and Cannons, J.L. (2009). SLAM receptors and SAP influence lymphocyte interactions, development and function. Nat. Rev. Immunol. *9*, 39–46.

Schwickert, T.A., Victora, G.D., Fooksman, D.R., Kamphorst, A.O., Mugnier, M.R., Gitlin, A.D., Dustin, M.L., and Nussenzweig, M.C. (2011). A dynamic T cell-limited checkpoint regulates affinity-dependent B cell entry into the germinal center. J. Exp. Med. *208*, 1243–1252.

Shan, Q., Zeng, Z., Xing, S., Li, F., Hartwig, S.M., Gullicksrud, J.A., Kurup, S.P., Van Braeckel-Budimir, N., Su, Y., Martin, M.D., et al. (2017). The transcription factor Runx3 guards cytotoxic $CD8^+$ effector T cells against deviation towards follicular helper T cell lineage. Nat. Immunol. *18*, 931–939.

Slack, E., Balmer, M.L., and Macpherson, A.J. (2014). B cells as a critical node in the microbiota-host immune system network. Immunol. Rev. *260*, 50–66.

Subramanian, A., Tamayo, P., Mootha, V.K., Mukherjee, S., Ebert, B.L., Gillette, M.A., Paulovich, A., Pomeroy, S.L., Golub, T.R., Lander, E.S., and Mesirov, J.P. (2005). Gene set enrichment analysis: a knowledge-based approach for interpreting genome-wide expression profiles. Proc. Natl. Acad. Sci. USA *102*, 15545–15550.

Victora, G.D., and Nussenzweig, M.C. (2012). Germinal centers. Annu. Rev. Immunol. *30*, 429–457.

Victora, G.D., Schwickert, T.A., Fooksman, D.R., Kamphorst, A.O., Meyer-Hermann, M., Dustin, M.L., and Nussenzweig, M.C. (2010). Germinal center dynamics revealed by multiphoton microscopy with a photoactivatable fluorescent reporter. Cell *143*, 592–605.

Vinuesa, C.G., and Cyster, J.G. (2011). How T cells earn the follicular rite of passage. Immunity *35*, 671–680.

Vinuesa, C.G., Linterman, M.A., Goodnow, C.C., and Randall, K.L. (2010). T cells and follicular dendritic cells in germinal center B-cell formation and selection. Immunol. Rev. *237*, 72–89.

von Boehmer, L., et al. (2016). Sequencing and cloning of antigen-specific antibodies from mouse memory B cells. Nat Protoc., 1908–1923.

Yaari, G., Uduman, M., and Kleinstein, S.H. (2012). Quantifying selection in high-throughput Immunoglobulin sequencing data sets. Nucleic Acids Res. *40*, e134.

Ye, J., Ma, N., Madden, T.L., and Ostell, J.M. (2013). IgBLAST: an immunoglobulin variable domain sequence analysis tool. Nucleic Acids Res. *41*, W34-40.

Yeap, L.S., Hwang, J.K., Du, Z., Meyers, R.M., Meng, F.L., Jakubauskaitė, A., Liu, M., Mani, V., Neuberg, D., Kepler, T.B., et al. (2015). Sequence-Intrinsic Mechanisms that Target AID Mutational Outcomes on Antibody Genes. Cell *163*, 1124–1137.

Zaretsky, I., Atrakchi, O., Mazor, R.D., Stoler-Barak, L., Biram, A., Feigelson, S.W., Gitlin, A.D., Engelhardt, B., and Shulman, Z. (2017). ICAMs support B cell interactions with T follicular helper cells and promote clonal selection. J. Exp. Med. *214*, 3435–3448.

Zhong, M.-C., and Veillette, A. (2013). Critical role of SAP in progression and reactivation but not maintenance of T cell-dependent humoral immunity. Mol. Cell. Biol. *33*, 1223–1232.

STAR★METHODS

KEY RESOURCES TABLE

REAGENT or RESOURCE	SOURCE	IDENTIFIER
Antibodies		
CD45R Monoclonal Antibody (RA3-6B2), APC-Alexa Fluor 750	Thermo Fisher Scientific	Cat# RM2627, RRID:AB_10371897
V500 Rat anti-Mouse CD45R/B220 (Clone RA3-6B2)	BD biosciences	Cat.#561226
CD45R (B220) Monoclonal Antibody (RA3-6B2), eFluor 450	Thermo Fisher Scientific	Cat# 48-0452-82, RRID:AB_1548761
Brilliant Violet 605 anti-mouse CD138 (Syndecan-1) antibody	BioLegend	Cat# 142531, RRID:AB_2715767
Anti-Mouse CD38 Alexa Fluor 700	Thermo Fisher Scientific	Cat# 56-0381-82, RRID:AB_657740
F4/80 Monoclonal Antibody (BM8), APC-eFluor 780, eBioscience	Thermo Fisher Scientific	Cat# 47-4801-80, RRID:AB_2637188
PE-Cy7 Hamster Anti-Mouse CD95 (Clone Jo2)	BD biosciences	Cat# 557653, RRID:AB_396768
Alexa Fluor® 647 anti-mouse/human GL7 Antigen	BioLegend	Cat# 144605, RRID:AB_2562184
FITC anti-mouse/human GL7 Antigen	BioLegend	Cat# 144604, RRID:AB_2561697
PerCP/Cy5.5 anti-mouse/human GL7 Antigen	BioLegend	Cat# 144609, RRID:AB_2562978
Biotin anti-mouse IgA	BioLegend	Cat# 407003, RRID:AB_315078
Mouse IgA Monoclonal Antibody (mA-6E1), PE	Thermo Fisher Scientific	Cat# 12-4204-81, RRID:AB_465916
Brilliant Violet 421 anti-mouse IgG1	BioLegend	Cat# 406615, RRID:AB_2562233
Mouse IgM Monoclonal Antibody (II/41), PerCP-eFluor 710	Thermo Fisher Scientific	Cat# 46-5790-82, RRID:AB_1834435
Streptavidin APC-eFluor 780 100 ug	Thermo Fisher Scientific	Cat# 47-4317-82, RRID:AB_10366688
Alexa Fluor® 488 anti-mouse CD4	BioLegend	Cat# 100423, RRID:AB_389302
CD4 Monoclonal Antibody (RM4-5), APC-eFluor 780	Thermo Fisher Scientific	Cat# 47-0042-80, RRID:AB_1272219
CD44 Monoclonal Antibody (IM7), PE	Thermo Fisher Scientific	Cat# A25999, RRID:AB_2536059
CD62L (L-Selectin) Monoclonal Antibody (MEL-14), Alexa Fluor 700	Thermo Fisher Scientific	Cat# 56-0621-82, RRID:AB_494003
CD8a Monoclonal Antibody (53-6.7), APC-eFluor 780	Thermo Fisher Scientific	Cat# 47-0081-82, RRID:AB_1272185
Ly-6G/Ly-6C Monoclonal Antibody (RB6-8C5), Super Bright 780	Thermo Fisher Scientific	Cat# 78-5931-82, RRID:AB_2744921
F4/80 Monoclonal Antibody (BM8), APC-eFluor 780	Thermo Fisher Scientific	Cat# 47-4801-80, RRID:AB_2637188
Pacific Blue anti-mouse CD62L	BioLegend	Cat# 104424, RRID:AB_493380
PE/Cy7 anti-mouse CD279 (PD-1)	BioLegend	Cat# 109110, RRID:AB_572017
Biotin anti-mouse CD185 (CXCR5)	BioLegend	Cat# 145509, RRID:AB_2562125
BCL6 Monoclonal Antibody (BCL-UP), PerCP-eFluor 710	Thermo Fisher Scientific	Cat# 46-9880-42, RRID:AB_11063697
Biotin anti-mouse CD275 (B7-RP1, ICOSL, B7H2)	BioLegend	Cat# 107403, RRID:AB_345259
BV421 Rat Anti-Mouse CD40	BD Biosciences	Cat# 562846, RRID:AB_2734767
Alexa Fluor® 488 anti-mouse CD54	BioLegend	Cat# 116111, RRID:AB_493494
Alexa Fluor(R) 647 anti-mouse CD102	BioLegend	Cat# 105612, RRID:AB_2122182
PE anti-mouse CD11a/CD18 (LFA-1)	BioLegend	Cat# 141006, RRID:AB_10694371
APC anti-mouse CD86	BioLegend	Cat# 105114, RRID:AB_313167
Brilliant Violet 421 anti-mouse CD184 (CXCR4)	BioLegend	Cat# 146511, RRID:AB_2562788
PE anti-mouse CD3	BioLegend	Cat# 100205, RRID:AB_312662
Anti-IgG2ab-VioBright FITC	Miltenyi Biotec	Cat# 130-104-579, RRID:AB_2661523
Goat polyclonal Secondary Antibody to Mouse IgG1 - heavy chain (HRP)	Abcam	Cat# ab97240, RRID:AB_10695944
Alexa Fluor® 647 anti-mouse IgD	BioLegend	Cat# 405707, RRID:AB_893529
InVivoMab anti-mouse CD4 (GK1.5)	Bio X Cell	Cat# BE0003-1, RRID:AB_1107636
Ultra-LEAF Purified anti-mouse CD8a (53-6.7)	BioLegend	Cat# 100746, RRID:AB_11147171

(Continued on next page)

Continued		
REAGENT or RESOURCE	SOURCE	IDENTIFIER
Bacterial and Virus Strains		
Recombinant influenza X31-GP33 virus	Lintermann laboratory	NA
LCMV-clone 13	Iannacone laboratory	NA
LCMV-Armstrong	Iannacone laboratory	NA
Chemicals, Peptides, and Recombinant Proteins		
Alexa Fluor® 647 Streptavidin	BioLegend	Cat# 405237
NP-OVAL (Ovalbumin)	Biosearch Technologies	Cat# 10643
Imject Alum Adjuvant	Thermo Fisher Scientific	Cat# 77161
Mouse IgA ELISA Quantitation Set	Bethyl Laboratories	Cat# E90-103
Hoechst 33342	Thermo Fisher Scientific	Cat# H3570
TRI Reagent	Sigma-Aldrich	Cat# T9424
Click-iT EdU Alexa Fluor 488 Flow Cytometry Assay Kit	Thermo Fisher Scientific	Cat# C10425
qScript cDNA Synthesis Kit	Quanta Bio	Cat# 95047
Qubit dsDNA HS Assay Kit	Thermo Fisher Scientific	Cat# Q32854
LightCycler® 480 SYBR Green I Master	Roche	Cat# 04707516001
Tissue-Tek® O.C.T. Compound	Sakura	Cat# 4583
Mounting medium	Sigma-Aldrich	Cat# M1289
Deposited Data		
BCR-seq of IgA^{+} GC B cells	This paper	ENA: PRJEB36003
RNA-seq of GC B cells	This paper	GEO: GSE142640
Experimental Models: Organisms/Strains		
WT: C57BL/6	Envigo	NA
AID^{Cre}: B6.129P2-*Aicda*$^{tm1(cre)Mnz}$/J	Jackson Laboratories	Cat# 007770 RRID:IMSR_JAX:007770
Rosa26$^{flox-stop-flox-tdTomato}$: B6.Cg-Gt(ROSA)26Sor$^{tm9(CAG-tdTomato)Hze}$/J	Jackson Laboratories	Cat# 007909 RRID:IMSR_JAX:007909
Blimp-1-YFP: B6.Cg-Tg(Prdm1-EYFP)1Mnz/J	Jackson Laboratories	Cat# 008828 RRID:IMSR_JAX:008828
TCRα^{KO}: B6.129S2-Tcra^{tm1Mom}/J	Jackson Laboratories	Cat# 002116 RRID:IMSR_JAX:002116
AID-GFP: C57BL/6-Tg(Aicda/EGFP)1Rcas/J	Nussenzweig laboratory	Cat# 018421 RRID:IMSR_JAX:018421
SAP^{KO}: B6.129S6-Sh2d1a^{tm1Pls}/J	Jackson Laboratories	Cat# 025754 RRID:IMSR_JAX:025754
Oligonucleotides		
See Table S1 for qPCR primer sequences	This paper	NA
Software and Algorithms		
Flowjo 10	Tree Star	https://www.flowjo.com/
Prism 5	Graphpad Software	https://www.graphpad.com/
Prism 7	Graphpad Software	https://www.graphpad.com/
BASELINe	Yaari et al., 2012	http://selection.med.yale.edu/baseline
MARS-seq Pipeline	Kohen et al., 2019	NA
Partek Genomics Suite Analysis software	Partek	https://www.partek.com/partek-genomics-suite/
GSEA	Subramanian et al., 2005; Mootha et al., 2003	http://software.broadinstitute.org/gsea/index.jsp
Imaris 9.1.2	Bitplane	https://imaris.oxinst.com/

LEAD CONTACT AND MATERIALS AVAILABILITY

Further information and requests for resources and reagents should be directed to and will be fulfilled by the Lead Contact, Ziv Shulman (ziv.shulman@weizmann.ac.il). This study did not generate new unique reagents.

EXPERIMENTAL MODEL AND SUBJECT DETAILS

Mice

Aicda^{Cre}, Rosa26$^{Stop\text{-}tdTomato}$, Sh2d1a$^{-/-}$ (SAP^{KO}), Blimp-1-YFP and TCR$\alpha^{-/-}$ (TCRα^{KO}) mice were purchased from the Jackson Laboratories. AID-GFP mice were provided by Prof. Michel Nussenzweig. SAP knockout mice (Sh2d1a$^{-/-}$) were previously described (Crotty et al., 2003). Sh2d1a$^{-/-}$ mice were bred with Aicda$^{Cre/+}$ Rosa26$^{Stop\text{-}tdTomato}$ and AID-GFP mice. Wild-type mice (C57BL/6) were purchased from Envigo. All mice were between the age of 8-12 weeks at the start of the experiment and no differences were observed between males and females. Mice were bred and housed in specific pathogen free conditions and all experiments were conducted under protocols approved by the Weizmann Institute Animal Care and Use Committee (IACUC).

Immunizations, treatments and viral infections

For NP-OVA immunizations, mice were injected with 25 μl PBS containing 10 μg NP16- OVA precipitated in alum (2:1) into the hind footpads. For influenza A virus infection experiments, mice were administered 10^4 plaque-forming units (PFU) of influenza A/Hong Kong/1/1968/x31 (x31) virus, 4×10^5 IU intranasally under inhalation anesthesia as previously described (Denton et al., 2019). For LCMV infection, mice were infected intravenously with $2x10^6$ or $2x10^5$ PFU of LCMV$_{cl13}$ or LCMV$_{arm}$, respectively (Sammicheli et al., 2016). For CD4$^+$ T cell depletion experiments, 200 μg of rat anti-mouse CD4 mAb (clone GK1.5, rIgG2b, BioXcell) or/and rat anti-mouse CD8 mAb (clone 53-6.7, rIgG2a, Biolegend) were injected intravenously 4 days prior to analysis. For Igh sequencing experiment, mice were injected with the depleting antibody every 4 days until analysis.

METHOD DETAILS

ELISA

Serum was collected from unimmunized mice, and IgG1 antibodies were detected by ELISA using anti-mouse IgG1-horseradish peroxidase. For IgA detection in the intestinal content, the small intestines were flushed with 2 mL PBS and collected into a tube. Samples were span down to remove feces and the supernatant was used for IgA detection by mouse IgA ELISA quantification set (Bethyl Laboratories).

In vivo EdU proliferation assay

For proliferation measurements, mice were injected intravenously with 2 mg of the nucleoside analog 5-ethynyl-2′-deoxyuridine (EdU) (Molecular Probes) in PBS. After 2.5 hours, the PPs were dissected and cells were then stained for surface antigens as described, followed by EdU detection using Click-iT EdU Alexa Fluor 488 Flow Cytometry Assay Kit (Molecular Probes) according to manufacturer's protocol.

Flow cytometry

Small intestines were excised and washed with ice cold PBS to remove fecal content. Peyer's patches were harvested and forced through a mesh into PBS containing 2% fetal calf serum and 1 mM EDTA. For blockade of Fc receptors, single cell suspensions were incubated with 2 μg/ml anti-16/32 (clone 93) for 5 min. Cells were washed and incubated with fluorescently labeled antibodies for 30 min. GC cells were gated as live/single, B220$^+$ CD38$^-$ FAS$^+$ or B220$^+$ GL-7$^+$ FAS$^+$. Tfh cells were gated as CD4$^+$ CD44$^+$ CD62L$^-$ PD-1$^+$ CXCR5$^+$. Intracellular staining for Bcl6 was performed using Foxp3 / Transcription Factor Staining Buffer Set (eBioscience) according to the manufacturer's instructions. Stained cell suspensions were analyzed using a CytoFlex flow cytometer (Beckman Coulter).

Quantitative PCR analysis

Total RNA was isolated using Trizol Reagent (Sigma-Aldrich) according to the manufacturer's instructions. Total RNA was subjected to cDNA synthesis using qScript synthesis kit (Quanta bio). cDNA concentration was assessed with Qbit, using the dsDNA high-sensitivity assay kit (Thermo Fisher Scientific). qPCR mix was prepared using SYBR green (Roche) with primers described in Table S1. Relative transcript expression was calculated using the ddCt method and all transcripts were normalized to HPRT.

Immunohistochemistry

Mediastinal lymph nodes were fixed with 4% paraformaldehyde fixative for 16 hours and then dehydrated in 30% sucrose overnight before being embedded in OCT (Tissue-Tek). OCT-embedded 10-mm cryostat sections were dehydrated in acetone prior to freezing. Sections were rehydrated in PBS and incubated with 1% SDS in PBS for 5 min. Sections were then blocked in PBS with 0.05% Tween-20 and 3% BSA for 1 hour prior to staining. Slides were stained with FITC conjugated anti-mouse GL-7 (diluted 1:100) and Alexa Fluor 647 conjugated anti-mouse IgD (1:200) (BioLegend) in 1%BSA in PBS/T overnight. Slides were washed and stained shortly with Hoechst (1:20,000) (Thermo Fisher Scientific). Sections were mounted with mounting medium (Sigma-Aldrich) and examined with a Zeiss LSM 880 confocal microscope. Imaging data were analyzed with Imaris 9.2 (Bitplane).

Image acquisition by TPLSM

Zeiss LSM 880 upright microscope fitted with Coherent Chameleon Vision laser was used for whole lymph node scan imaging experiments. Images were acquired with a femtosecond-pulsed two-photon laser tuned to 940 nm. The microscope was fitted with a filter cube containing 565 LPXR to split the emission to a PMT detector (with a 579-631 nm filter for tdTomato fluorescence) and to an additional 505 LPXR mirror to further split the emission to 2 GaAsp detectors (with a 500-550nm filter for GFP fluorescence). Tile images were acquired as a 120-180 μm Z stacks with 10 μm steps between each Z-plane. The zoom was set to 1.5, and pictures were acquired at 512 × 512 x-y resolution.

Image analysis

In the whole lymph node scan images, quantification of GC size was performed using Imaris software (Bitplane). Each GC structure was segmented separately using the Imaris 3D surface object and area measurements were used.

Single cell Igh sequencing

Single PP was harvested from WT and SAP- deficient mice and processed for flow cytometry analysis. Cell suspensions were gated as B220$^+$ GL-7$^+$ FAS$^+$ IgA$^+$ representing GC cells (Figure S5). Cell sorting was performed using a FACS Aria II cell sorter (BD Bioscience). For total VDJ sequencing of Igα heavy chains, cells were sorted into 96 well plates containing lysis buffer (PBS containing 3 U/μl RNAsin, 10 mM dithiothreitol –DTT). cDNA was purified using random primers (NEB) as previously described (von Boehmer, 2016). Igα heavy chain sequence was amplified twice using primers for the Igα constant region (5′-ATCAGGCAGCCGATTATCAC-3′ for the first reaction and 5′-GAGGTGCAGCTGCAGGAGTCTGG-3′ for the second reaction) (Lindner et al., 2015) together with a mix of primers for the variable region (Ho et al., 2016). The PCR products were sequenced and analyzed for CDR3 using web-based IgBlast and IMGT tools. Sequence alignment was performed using SnapGene software (GSL Biotech). For schematic representation of the results, sequences derived from single cells were clustered according to their CDR3 region and presented as percent of the total sequenced cells. Primer-derived mutations were excluded from the analysis.

Igh sequence analysis

Ig Fasta sequences were aligned against the IMGT mouse heavy chain gene database (Sep. 2017) using NCBI IgBlast (version 1.7.0) (Ye et al., 2013). Post processing of IgBlast output, and clonal clustering were performed using Change-O v0.3.7 (https://changeo.readthedocs.io/) (Gupta et al., 2015), Alakazam v0.2.8 (https://alakazam.readthedocs.io), SHazaM v0.1.8 (https://shazam.readthedocs.io), and custom scripts within the R statistical computing environment, as follows. V(D)J sequences were assigned to clonal groups by partitioning sequences based on identity of IGHV gene annotations, IGHJ gene annotations, and junction region lengths. Within these groups, sequences differing from one another by a hamming distance of 0.2 within the junction region were defined as clones by single-linkage clustering. Distances were measured and normalized by the length of the junction region. The clonal distance threshold was determined by manual inspection to identify the local minima between the two modes of the within-sample bimodal distance-to nearest histogram. Full-length germline sequences were reconstructed for each clonal cluster with D segment and N/P regions masked (replaced with Ns), with any ambiguous gene assignments within clonal groups resolved by the majority rule. Lineage trees were constructed for each clone having at least two unique sequences using PHYLIP (v3.695) (Felsenstein, 2005) and Alakazam. Selection quantification was calculated using BASELINe's local test (Yaari et al., 2012).

RNA sequencing and gene set enrichment analysis

PPs were harvested from WT and SAP^{KO} mice and processed for flow cytometry analysis. Cell suspensions were stained for Dump-(CD4, CD8, Gr-1, F4/80) and B220$^+$ CD138$^-$ GL-7$^+$ FAS$^+$, representing GC B cells. Cell sorting was performed using a FACS Aria cell sorter (BD Bioscience). 5-10x10^3 cells were sorted into 60μl of lysis/binding buffer (Life Technologies). mRNA was captured with 12 mL of oligo(dT) Dynabeads (Life Technologies), washed, and eluted at 85°C with 10 μl of 10 mM Tris-Cl (pH 7.5). MARS-seq was used as described (Jaitin et al., 2014) to produce expression libraries with six replicates per strain. MARS-seq analysis was done using the UTAP transcriptome analysis pipeline (Kohen et al., 2019) of the Weizmann Institute Bioinformatics Unit. Reads were trimmed using Cutadapt (Martin, 2011) and mapped to the Mus_musculus genome (UCSC mm10) using STAR (Dobin et al., 2013) v2.4.2a with default parameters. The pipeline quantifies the genes annotated in RefSeq (extended by 1000 bases toward the 5′ edge and 100 bases in the 3′ direction). Counting of sequenced reads was done using htseq-count (Anders et al., 2015) (union mode). Genes having minimum of 5 UMI-corrected reads in at least one sample, were considered. Normalization of the counts and differential expression analysis was performed using DESeq2 (Love et al., 2014) with the parameters: betaPrior = True, cooksCutoff = FALSE, independentFiltering = FALSE. Raw P values were adjusted for multiple testing using the procedure of Benjamini and Hochberg. The threshold for significant differential expression was: padj $\leq$ 0.05, |log2FoldChange| > = 1 and BaseMean > = 5. Gene Set Enrichment Analysis (GSEA) was performed using GSEA 3.0 with the GSEAPreranked tool (Mootha et al., 2003; Subramanian et al., 2005). Gene names were converted to human gene symbols, and ran with default parameters for genes with BaseMean > = 5. The Molecular Signature Database (MSigDB) hallmark gene sets was used to perform pathway enrichment analysis using a hypergeometric distribution and limiting the output to the top 1000 gene sets.

QUANTIFICATION AND STATISTICAL ANALYSIS

Students' t tests or one-way ANOVA with bonferroni posttest were used for statistical analysis and indicated in figure legends. Unless otherwise indicated, the data in figures were displayed as the mean ± SEM and n represents number of mice analyzed. p values are denoted in figures by: ns, not significant; *, $p < 0.05$; **, $p < 0.01$; ***, $p < 0.0001$. Statistical measurement was determined using Graphpad Prism Version 5.0.

DATA AND CODE AVAILABILITY

The accession number for all BCR sequencing data reported in this paper isENA: PRJEB36003. The accession number for all RNA-seq data reported in this paper is GEO: GSE142640. Custom scripts used for data analysis are available upon request. All other data are available in the main text or the supplementary materials.

Cell Reports, Volume *30*

Supplemental Information

B Cell Diversification Is Uncoupled from SAP-Mediated Selection Forces in Chronic Germinal Centers within Peyer's Patches

Adi Biram, Eitan Winter, Alice E. Denton, Irina Zaretsky, Bareket Dassa, Mats Bemark, Michelle A. Linterman, Gur Yaari, and Ziv Shulman

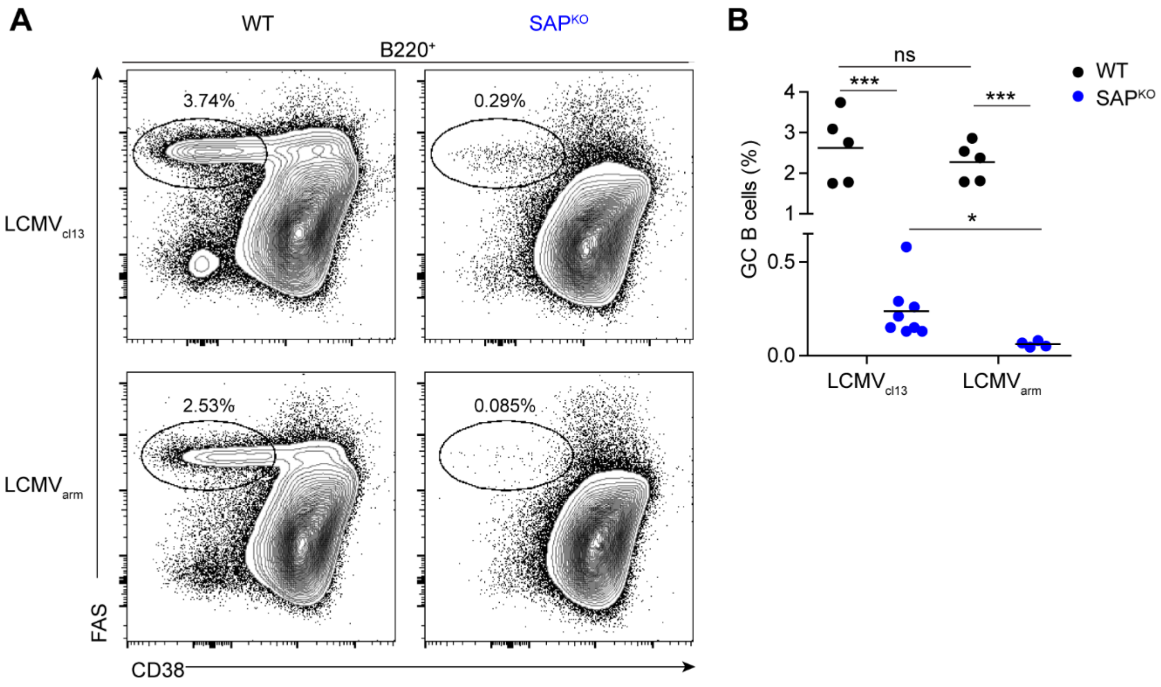

Figure S1. GC formation in the spleen in response to acute and chronic LCMV infection, Related to figure 2. (A) Representative flow cytometry plots showing GC frequency ($B220^+$ FAS^+ $CD38^-$) in the spleen of WT and SAP^{KO} mice, 30 days following intravenous infection with $LCMV_{cl13}$ or $LCMV_{arm}$. **(B)** Quantification of GC frequencies analyzed as in (A). * P<0.05, *** P<0.0001, one-way ANOVA with Bonferroni posttest. ns, not significant.

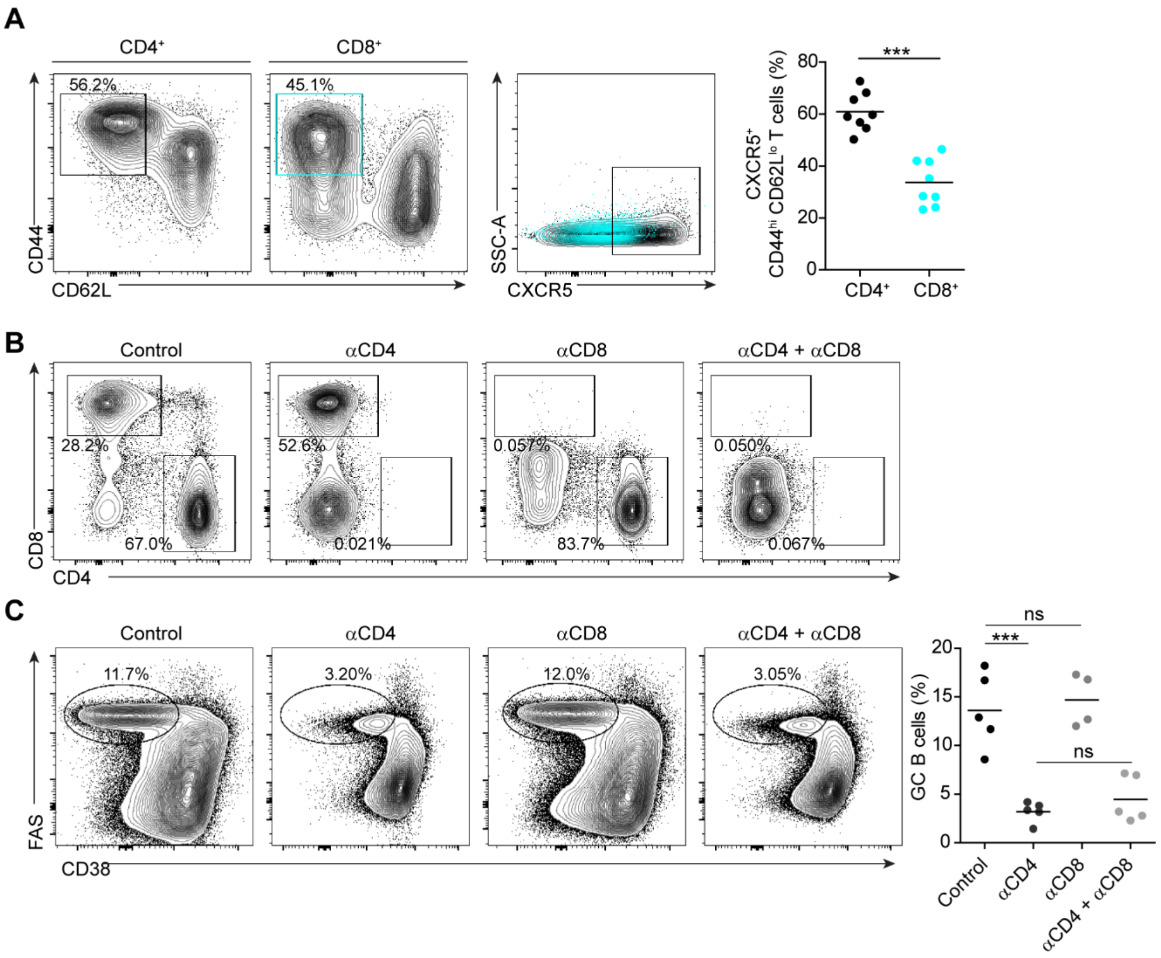

Figure S2. CD8 T cell depletion does not affect GC frequency, Related to figure 3. (A) Representative flow cytometry plots showing gating on activated T cells (CD44^{hi} CD62L^{lo}) and CXCR5 expression on CD4^{+} or CD8^{+} activated T cells. Quantification is summarized in the graph. **(B)** Representative flow cytometry plots showing the CD4^{+} and CD8^{+} T cell populations (gated on CD3^{+}) following intravenous administration of 200µg or 100µg depleting antibodies for CD4 and CD8 depletion, respectively. **(C)** Representative flow cytometry plots showing GC frequency following the different treatments as described in B. Quantification is shown in the graph. *** P<0.0001, two-tailed Student's t test in (A). One-way ANOVA with Bonferroni posttest in (C). ns, not significant.

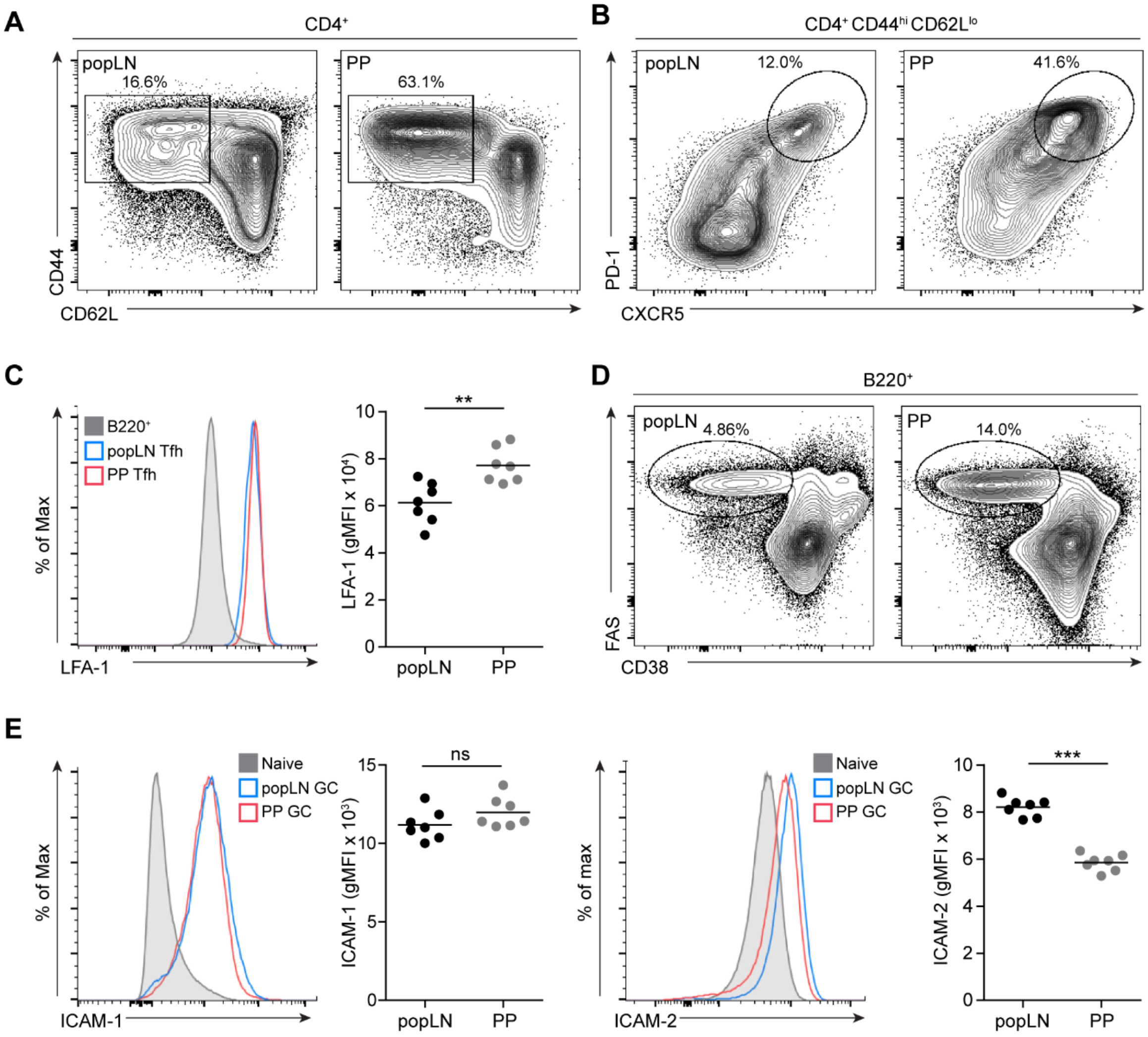

Figure S3. LFA-1 and ICAM expression on Tfh and GC B cells in popLNs and PPs, Related to figure 3. (A) Representative flow cytometry plots showing gating of activated T cells (CD44^{hi} CD62L^{lo}) in popLN and PP of WT mice, 7 days after NP-OVA intra-footpad immunization. **(B)** Representative flow cytometry plots showing Tfh cell population (CXCR5^{hi} PD-1^{hi}) in T cells gated as in (A). **(C)** Representative histogram showing LFA-1 expression in Tfh cells gated as in (B). Graph shows quantification. **(D)** Representative flow cytometry plots showing gating of GC B cells (B220^{+} FAS^{+} CD38^{-}) in PPs and immunized popLNs, as described in (A). **(E)** Representative histograms and graphs showing ICAM-1 and ICAM-2 expression in GC B cells gated as in (D). ** P<0.001, *** P<0.0001, two-tailed Student's t test; ns, not significant.

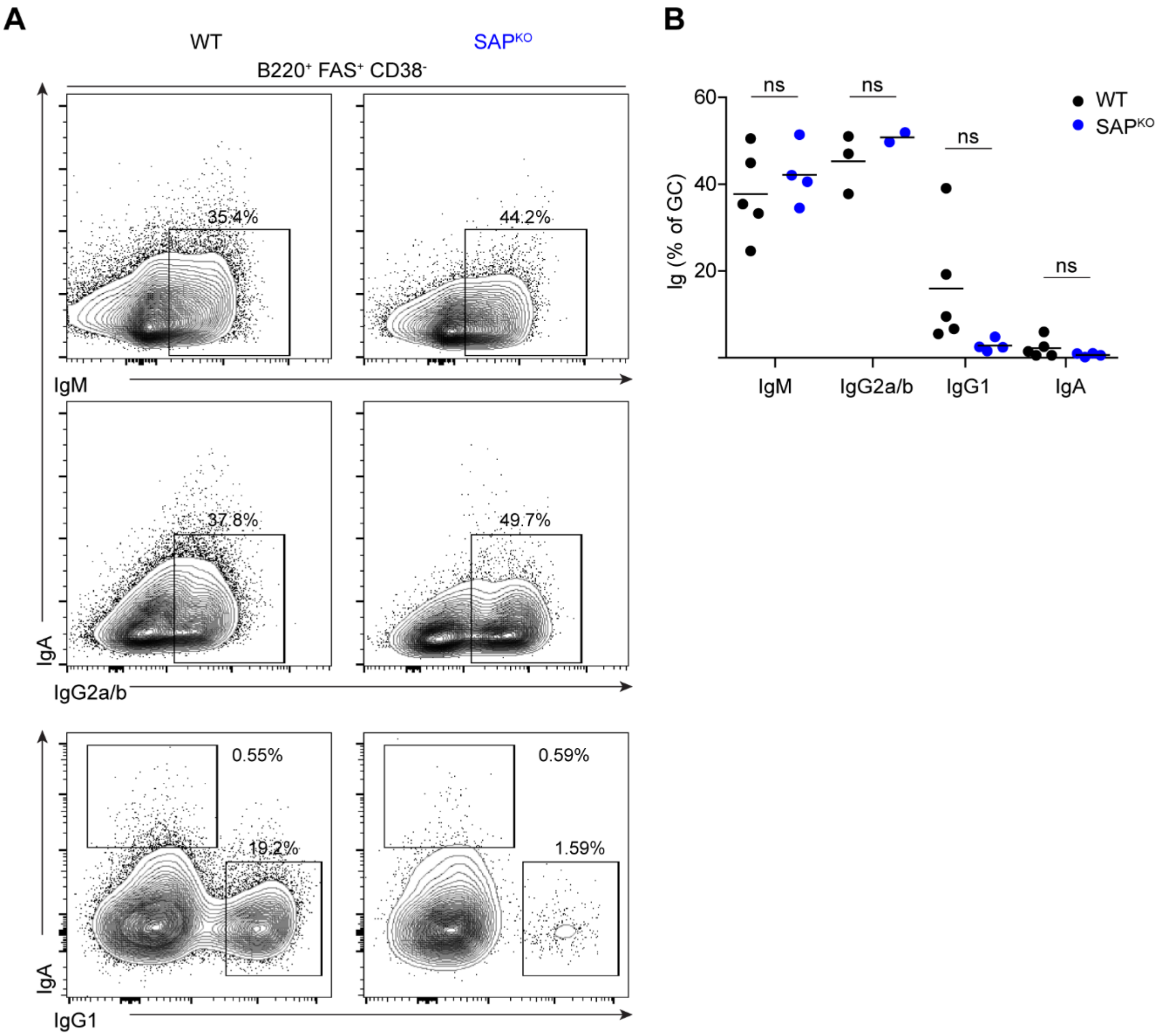

Figure S4. Ig isotypes of GC B cells in the mediastinal LN of SAP^{KO} mice following influenza infection, Related to figure 2. (A,B) Representative flow cytometry plots (A) and summary graph (B) showing IgM, IgG2a/b, IgA and IgG1 staining of germinal center B cells (FAS^{+} CD38^{-}) derived from mediastinal LNs of influenza (x31) infected WT and SAP^{KO} mice, 14 days after intranasal infection. One-way ANOVA with Bonferroni posttest; ns, not significant.

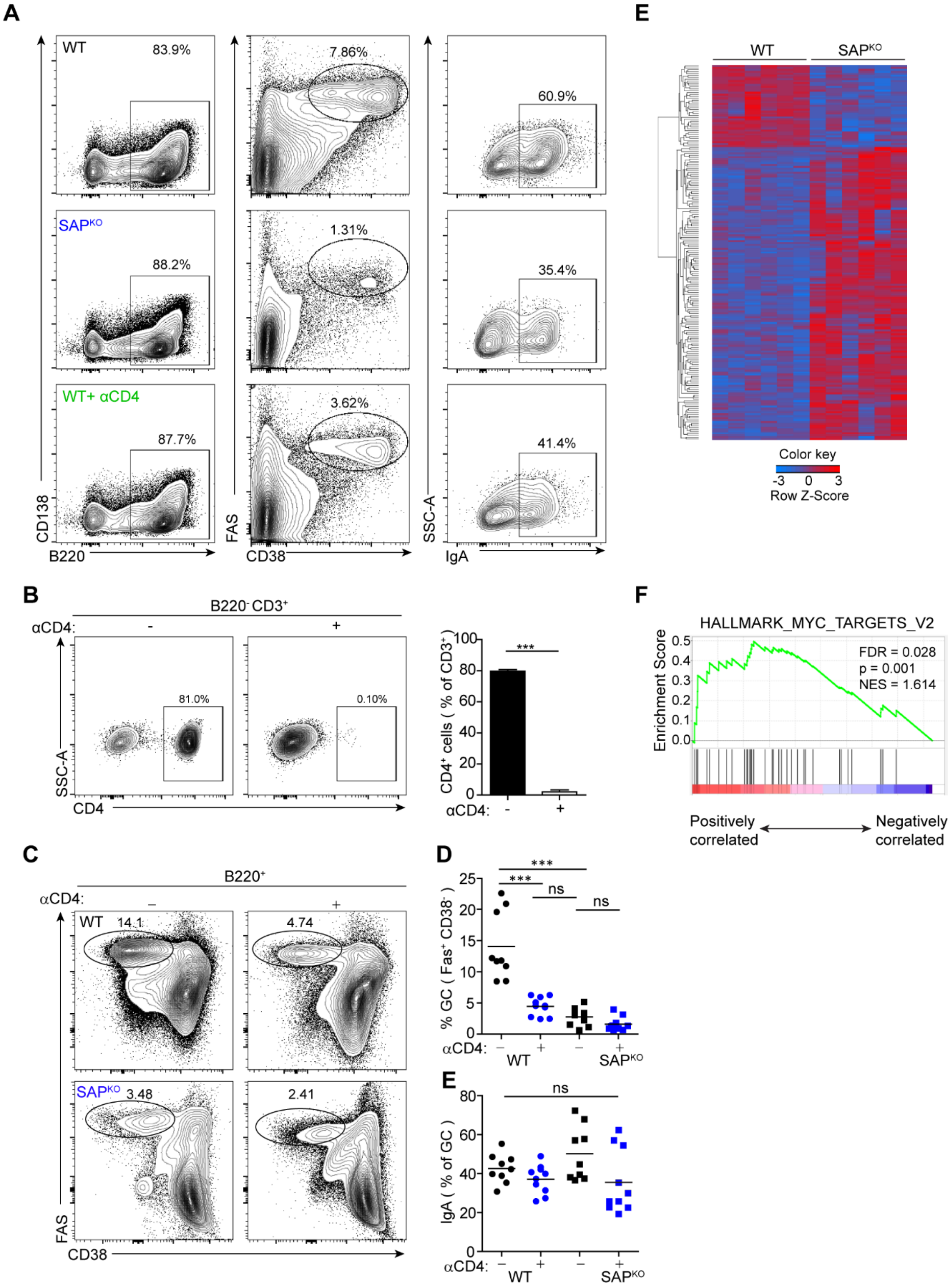
A
WT
83.9%
7.86%
60.9%
SAPKO
88.2%
1.31%
35.4%
WT+ αCD4
87.7%
3.62%
41.4%
CD138
B220
FAS
CD38
SSC-A
IgA
E
WT
SAPKO
Color key
-3 0 3
Row Z-Score
B
B220- CD3+
αCD4: - +
81.0%
0.10%
SSC-A
CD4
CD4+ cells (% of CD3+)
100
80
60
40
20
0
***
αCD4: - +
F
HALLMARK_MYC_TARGETS_V2
Enrichment Score
0.5
0.4
0.3
0.2
0.1
0.0
FDR = 0.028
p = 0.001
NES = 1.614
Positively correlated
Negatively correlated
C
B220+
αCD4: – +
WT 14.1
4.74
SAPKO 3.48
2.41
FAS
CD38
D
% GC (Fas+ CD38-)
25
20
15
10
5
0
***
***
ns
ns
αCD4: – + – +
WT
SAPKO
E
IgA (% of GC)
80
60
40
20
0
ns
αCD4: – + – +
WT
SAPKO

Figure S5. Gating strategy for PP IgA GC single-cell sorting for *Igh* sequencing, T cell depletion and GC B cell transcriptomic analysis, Related to figure 5. (A) Representative flow cytometry plots showing the strategy for single-cell sorting of $B220^{+}$ FAS^{+} $CD38^{-}$ IgA^{+} cells from a single PP of WT, SAP-deficient, and [WT + αCD4] mice. [WT + αCD4] mice were treated with an αCD4 depleting antibody for 14 days. **(B)** Representative flow cytometry plots showing the $CD4^{+}$ cell population in PPs of WT mice treated with 200μg αCD4 depleting antibody, 4 days after antibody administration. Graph shows quantification of $CD4^{+}$ cell number as in (A). **(C)** Representative flow cytometry plots showing GC frequency in WT and SAP-deficient PPs following T cell depletion. **(D)** Quantification of GC frequency, as presented in (C). **(E)** Frequency of IgA class switch recombination in GC B cells of WT and SAP-deficient PPs following αCD4 treatment. Data are pooled from three independent experiments with three mice in each experiment. Each dot represents a single mouse; line represents the mean. * $P<0.05$, *** $P<0.0001$, one way ANOVA with Bonferroni posttest in (D,E) and two-tailed Student's t test in (B). ns, not significant. **(F)** Heat map showing expression levels of differentially expressed genes (n=6 in each group), as analyzed by UTAP pipeline using a cutoff of: padj <= 0.05, |log2FoldChange| >= 1, and BaseMean >= 5. Expression levels of genes (log2 normalized, rld) were scaled and clustered by Hierarchical clustering (Euclidean, average linkage) and visualized using Partek Genomics Suite. **(G)** Gene set enrichment plot for Myc pathway target genes from the hallmark gene sets. FDR, Flase discovery rate; p, p-value; NES, normalized enrichment score. In H,I, six mice were used in each group (n=6).

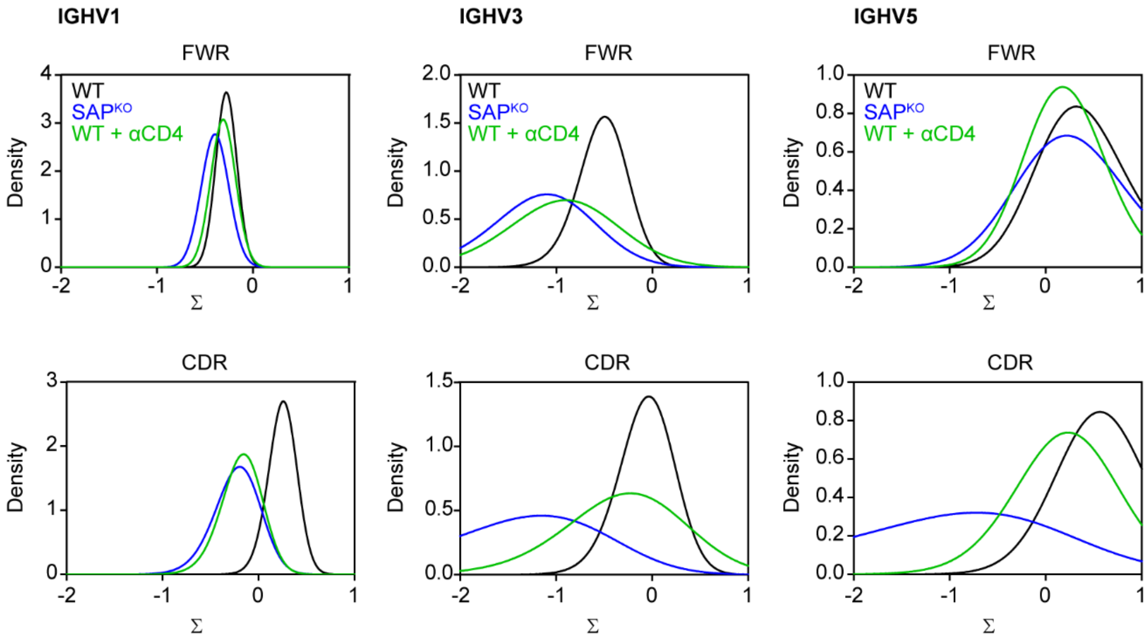

Figure S6. Estimation of selection in single V families shared among WT and SAP^{KO} Peyer's patch germinal center B cells, Related to figure 5. Graphs showing the density of selection strength for variable sequences of IGHV1, IGHV3 and IGHV5 within the framework (FWR) or complementary determining region (CDR) that were detected in WT, SAP^{KO} and αCD4 treated mice. Selection was estimated using BASELINe focused test with the MK RS5NF mutability model, FDR<0.05.

Primer name	Primer sequence 5'-3'
IL4R- F	GAGTGGAGTCCTAGCATCACG
IL4R-R	CAGTGGAAGGCGCTGTATC
IL21R-F	GGAGTGACCCCGTCATCTT
IL21R-R	AGGAGCAGCAGCATGTGAG
Stat3-F	GTTCCTGGCACCTTGGATT
Stat3-R	CAACGTGGCATGTGACTCTT
Stat6-F	GTCCATGAGTGTACTGCCATCT
Stat6-R	GGCATGGTTATCTGGCTCAT
Bcl6-F	GAACTGTATGCAGATTCCAGTCA
Bcl6-R	ACTGTCCTCTTGTAATCCTTCCA
HPRT-F	GTTAAGCAGTACAGCCCCAAA
HPRT-R	AGGGCATATCCAACAACAAACTT

Table S1. List of primers used for qPCR, Related to figure 4.
